# Metabolic activities and molecular investigations of the ameliorative impact of some growth biostimulators on chilling-stressed coriander (*Coriandrum sativum* L.) plant

**DOI:** 10.1186/s12870-021-03021-6

**Published:** 2021-08-07

**Authors:** Raifa A. Hassanein, Omaima S. Hussein, Amal F. Abdelkader, Iman A. Farag, Yousra E. Hassan, Mohamed Ibrahim

**Affiliations:** 1 Department of Botany, Faculty of Science, Ain Shams University, Cairo, 11355 Egypt; 2 Department of Natural Products, National Center for Radiation Research and Technology, Atomic Energy Authority, P.O. 29, Cairo, Nasr City Egypt

**Keywords:** Low temperature stress, *Coriandrum sativum* L., Growth biostimulators, Growth hormones, Antioxidants, RuBisCO complex integrity, HDN-PAGE

## Abstract

**Background:**

Priming of seed prior chilling is regarded as one of the methods to promote seeds germination, whole plant growth, and yield components. The application of biostimulants was reported as beneficial for protecting many plants from biotic or abiotic stresses. Their value was as important to be involved in improving the growth parameters of plants. Also, they were practiced in the regulation of various metabolic pathways to enhance acclimation and tolerance in coriander against chilling stress. To our knowledge, little is deciphered about the molecular mechanisms underpinning the ameliorative impact of biostimulants in the context of understanding the link and overlap between improved morphological characters, induced metabolic processes, and upregulated gene expression. In this study, the ameliorative effect(s) of potassium silicate, HA, and gamma radiation on acclimation of coriander to tolerate chilling stress was evaluated by integrating the data of growth, yield, physiological and molecular aspects.

**Results:**

Plant growth, yield components, and metabolic activities were generally diminished in chilling-stressed coriander plants. On the other hand, levels of ABA and soluble sugars were increased. Alleviation treatment by humic acid, followed by silicate and gamma irradiation, has notably promoted plant growth parameters and yield components in chilling-stressed coriander plants. This improvement was concomitant with a significant increase in phytohormones, photosynthetic pigments, carbohydrate contents, antioxidants defense system, and induction of large subunit of RuBisCO enzyme production. The assembly of Toc complex subunits was maintained, and even their expression was stimulated (especially Toc75 and Toc 34) upon alleviation of the chilling stress by applied biostimulators. Collectively, humic acid was the best the element to alleviate the adverse effects of chilling stress on growth and productivity of coriander.

**Conclusions:**

It could be suggested that the inducing effect of the pretreatments on hormonal balance triggered an increase in IAA + GA_3_/ABA hormonal ratio. This ratio could be linked and engaged with the protection of cellular metabolic activities from chilling injury against the whole plant life cycle. Therefore, it was speculated that seed priming in humic acid is a powerful technique that can benefit the chilled along with non-chilled plants and sustain the economic importance of coriander plant productivity.

**Supplementary Information:**

The online version contains supplementary material available at 10.1186/s12870-021-03021-6.

## Background

Generally, chilling has been defined as that under low atmospheric temperatures no ice formed inside plant tissues. It has been previously reported that plant species subjected to low temperature emerged as one of the serious problems. This problem was reported previously by Wang et al. [99] in tropical and subtropical plants due to a sudden change in temperature. Chilling has a serious impact on the growth and production of commercial crop plants marked as sensitive to chilling like tomato, maize, cotton, pepper, soybean, rice, and affects tropical and subtropical fruits like bananas, papayas, mangoes, grapes, and oranges [86]. Furthermore, low-temperature results in a physiological disturbance known as chilling injury. Various plant developmental and physiological processes (like crop growth, cell division, photosynthesis, water transport, lipids, metabolites, and yield) are negatively affected by this injury [32, 59].

Coriander (*Coriandrum sativum* L.) is a Mediterranean famous herb that belongs to family Apiaceae (Umbelliferae) and characterized by its essential oils used in food industries. Also, coriander is considered as an essential ingredient in curry powder, pharmaceutical and medicinal industry, and cosmetics. Coriander is also well known for its antioxidant, anti-diabetic, anti-mutagenic, anti-anxiety and antimicrobial activity along with analgesic and hormone balancing effect. Furthermore, coriander is famous by containing many essential oil active compounds primarily monoterpenes, pinene, limpene, ý-terpinene, p-cymene, borenol, citronellol, camphor, geraniol, coriandrin, dihydrocoriandrin, coriandrons A-E, and, flavonoids. These components help in removing toxic mineral residues such as mercury and lead [54]. Coriander seeds, leaves, and roots are edible, possessing light and fresh distinct flavor. Fresh leaves and ripe fruits are mainly used for culinary purposes. The plant leaves are rich source of vitamins, while seeds are rich in polyphenols and essential oils [79]. The fruit contains 50% linalool composition used in pharmaceuticals (as good source of α-tocopherol and vitamin A), in cosmetic and hygienic industries, and in food and drug industries [79]. The previously mentioned coriander benefits have prompted us to focus our study on this valuable herb particularly the influence of low temperature (chilling) environmental factor on the coriander productivity.

Recently, regulation of the metabolic pathways was practiced in application of biostimulants, such as 2,4-dichlorophenoxyacetic acid exploited as stimulant in mango fruits, to enhance acclimation and tolerance in coriander subjected to chilling stress [98]. The silicon effect was reported as beneficial for protecting many plants from biotic or abiotic stresses [60]. Many investigations of primed plants with silicon have recorded greater membrane stability index under stress [56]. The value of potassium silicate was as important a nutritional supplement of both silicon and potassium, which are involved in improving the morphological characters of plants [25]. Humic acid (HA) is a derived acid from soil organic matter and originated from plants, microbes, carbohydrates, proteins, and lignin. HA is the major component of humic substance and is extractable in alkali soil media [96]. In addition, HA possess a powerful impact on improving soil fertility and facilitating root uptake by regulating their function and structure under normal or abiotic stress [16, 96]. The chemical structure of HA enhances chelation of soil minerals and increased acquisition of nutrients by plants [73]. Previous studies have demonstrated that HA derivatives get firmly attached to the root, aggregate on the cell wall, and solubilize quickly in the cell cytoplasm within few hours of treatment before moving upwards to the shoot [16]. Gamma radiation is also known as ionizing radiation that reacts with atoms and molecules inside the cells to produce free radicals. However, production of free radicals is dependent on the irradiation dose and likely causes damage or modification of components in plants, and ultimately affects the morphology, physiology, anatomy, and biochemistry of plants [7]. As a result, gamma alters photosynthesis, expansion of thylakoid membrane, accumulation of phenolic compounds, and variation of the antioxidative system [7]. It was reported that previously fertilized rice with silicon has grown better after exposure to gamma rays [61, 62].

Moreover, medicinal plants subjected to 50-Gy gamma irradiation had the maximal beneficial effects on stress acclimation, improvements in germination and growth/yield parameters, and active ingredients enhancement [6, 22, 89]. In addition, gamma irradiation was used for decontamination in medicinal plants [28, 35]. In the same context, application of low doses of gamma radiation (20–60 Gy) on chilled-primed *Apium graveolence* (L.) seeds, either at room temperature or at 5 °C, were effective in alleviating chilling stress by stimulating celery growth and proliferation [26]. Hereby, the aim of this study was to evaluate the ameliorative effect(s) of potassium silicate, HA, and gamma radiation on acclimation of coriander to tolerate chilling stress by recording the data of growth, yield, physiological and molecular aspects.

## Results

For sake of clarity and concise focus throughout showing the obtained results, the percentage of increase/decrease was calculated from the statistically analyzed represented data in the shown tables. The percentage of increase/decrease was calculated as an increase/decrease percentage value in accordance with the control value. This percentage value was calculated by subtracting the value of control reading from the reading value of any physiological treatment, then the result was divided by the reading of control value, and finally, the result is multiplied by 100. The experimental protocol is presented and listed in Table 1.

**Table 1:** Experimental protocol of coriander seeds primed using tap water, solutions of potassium silicate (80 mM) or humic acid (50 mg. L^− 1^) or irradiated by (50 Gy) γ-rays

No.	Treatment	Seed treatment	Seed protocol prior sowing
1	**Control**	Primed in water, non-chilled	16 h in non-chilled water 20°C ±2
2	**Chilling (6 °C ± 0.5)**	Primed in water, chilled	16 h in chilled water 6°C ±0.5
3	**Pot. silicate (80 mM)**	primed, non-chilled	16 h in non-chilled pot. Silicate
4	**Humic acid (50 mg l**^**− 1**^**)**	primed, non-chilled	16 h in non-chilled HA
5	**γ- rays (50 Gy)**	primed, non-chilled	16 h in non-chilled water and γ-irradiated seeds
6	**Chilling + Pot. silicate**	primed, chilled	16 h in chilled pot. Silicate
7	**Chilling + Humic acid**	primed, chilled	16 h in chilled HA
8	**Chilling + γ- rays**	primed, chilled	16 h in chilled water and γ-irradiated seeds

### Growth parameters

When compared with non-treated coriander plants, chilling stress caused a significant inhibition in all growth parameters (shoot and root lengths, fresh and dry weights of shoot and root, number of leaves/plant, number of branches/plant, leaves area/plant, and no. of inflorescences/plant) throughout experimental duration (Table 2). Generally, all growth parameters were stimulated by soaking seeds in potassium silicate, HA or exposed to γ-rays as compared with control and chilling-stressed coriander plants at the vegetative stage (Table 2). The most effective treatment was HA alone in both control and stress alleviated samples. At the flowering stage (Fig. S1 and Table 3), chilling stressed and alleviated coriander samples by HA treatment have recorded a significant increase in growth parameters (full length figure is attached as Fig. S1) evaluated by 29.03, 8.94, 91.6, 208.5, 216.1, 178.8, 132.3, 100, 402.9, and 80% respectively, more than chilling stressed samples (Table 3). The same parameters were increased over their corresponding control plants by 12.5, 15.6%, 56.5, 103.9, 218.4, 68.3%, 34.4, 36.4, 82.7, 28.6% for shoot length, root length, fresh and dry weights of shoot and root, number of leaves per plant, number of branches per plant, leaves area per plant, and no. of inflorescences per plant, respectively.

**Table 2:** Impact of alleviation treatments on growth parameters of chilling-stressed coriander plants at the vegetative stage. Coriander control plants and chilling-stressed ones were subjected to pot. Silicate (80 mM), HA (50 mgl^− 1^) or γ-radiation (50 Gy). The shown data was extracted by using 3 biological and 3 technical replicates. Each biological replicate is comprised of 10 plants (one pot). The mean value of each biological replicate represents the value of one technical replicate. The readings of the 3 technical replicates were recorded. The mean value of the technical replicates was used to calculate ±SE. Also, the least significant differences (LSD) at 5% level were calculated to compare the means of different treatments according to Snedecor and Cochran [92]. The values with the same letter are not significantly different (P<0.05). The raw data set of the technical replicates was attached as a supplementary file

Growth Parameters	Shoot length (cm)	No. of leaves/ plant	No. of branches/plant	Leaves area/plant (cm)^2^	F. wt. of shoot (g)	D. wt. of shoot (g)	Root length (cm)	F. wt. of root (g)	D. wt. of root (g)
Treatment									
Control	11.57 ± 0.23^d^	16.00 ± 0.57^e^	5.67 ± 0.33^a^	30.53 ± 0.34^e^	0.55 ± 0.06 ^e^	0.09 ± 0.004^bc^	6.43 ± 0.47^bc^	0.046 ± 0.005^f^	0.012 ± 0.001^c^
Chilling (6°C ± 0.5)	10.23 ± 0.14^e^	10.67 ± 0.88^f^	3.67 ± 0.33^b^	14.28 ± 0.7^f^	0.28 ± 0.02^e^	0.04 ± 0.003^c^	6.20 ± 0.15^c^	0.032 ± 0.002^g^	0.006 ± 0.0005^d^
Pot. silicate (80 mM)	17.40 ± 0.3^a^	27.67 ± 1.4^cd^	6.00 ± 0.57^a^	94.27 ± 0.83^b^	0.74 ± 0.08^de^	0.13 ± 0.003^bc^	6.63 ± 0.3^bc^	0.064 ± 0.002^e^	0.016 ± 0.002^c^
Humic acid (50 mg l^−1^)	18.10 ± 0.5^a^	34.33 ± 0.9 ^a^	7.00 ± 0.57^a^	106.12 ± 2^a^	2.034 ± 0.014^a^	0.21 ± 0.002^ab^	7.73 ± 0.08^a^	0.131 ± 0.006^a^	0.024 ± 0.0004^a^
γ- rays (50 Gy)	17.57 ± 0.23^a^	32.67 ± 1.5^ab^	7.00 ± 0.57^a^	89.64 ± 1.4^b^	1.18 ± 0.16 ^bc^	0.20 ± 0.008^ab^	7.37 ± 0.4^ab^	0.126 ± 0.01^a^	0.022 ± 0.003^a^
Chilling + Pot. silicate	14.33 ± 0.44^c^	30.00 ± 0.57d	6.33 ± 0.66^a^	57.39 ± 1.1^d^	1.22 ± 0.14^bc^	0.30 ± 0.11^bc^	7.40 ± 0.3^ab^	0.089 ± 0.006^d^	0.014 ± 0.0012^c^
Chilling + Humic acid	15.77 ± 0.15^b^	23.67 ± 2.9^bc^	6.00 ± 0.67^a^	71.82 ± 1.5^c^	0.97 ± 0.013 ^b^	0.12 ± 0.003^a^	7.97 ± 0.4^a^	0.106 ± 0.004^b^	0.022 ± 0.0004^a^
Chilling + γ- rays	15.50 ± 0.28^b^	25.00 ± 0.57^d^	7.00 ± 0.57^a^	59.34 ± 3.3^d^	1.23 ± 0.05^cd^	0.17 ± 0.007^abc^	6.67 ± 0.24^bc^	0.096 ± 0.005^c^	0.0164 ± 0.0015^bc^
LSD at 0.05	**0.877**	**4.227**	**1.598**	**4.744**	**0.2597**	**0.136**	**0.994**	**0.0055**	**0.0055**

**Table 3:** Impact of alleviation treatments on growth parameters of chilling-stressed coriander plants at the flowering stage. Coriander control plants and chilling-stressed ones were subjected to pot. Silicate (80 mM), HA (50 mgl^−1^) or γ-radiation (50 Gy). The shown data was extracted by using 3 biological and 3 technical replicates. Each biological replicate is comprised of 10 plants (one pot). The mean value of each biological replicate represents the value of one technical replicate. The readings of the 3 technical replicates were recorded. The mean value of the technical replicates was used to calculate ±SE. Also, the least significant differences (LSD) at 5% level were calculated to compare the means of different treatments according to Snedecor and Cochran [92]. The values with the same letter are not significantly different (P<0.05). The raw data set of the technical replicates was attached as a supplementary file. By being the best alleviation element against the chilling stress, the percentage of increase (inc.) in all measurements, triggered by HA application, was further investigated. This percentage was calculated by subtracting the value of control/chillied reading from the reading value of any physiological treatment, then the result was divided by the reading of control value, and finally, the result is multiplied by 100

Growth Parameters	Shoot length (cm)	No. of leaves/ plant	No. of branches /plant	Leaves area/plant (cm)^2^	F. wt. of root (g)	D. wt. of shoot (g)	Root length (cm)	F. wt. of shoot	D. wt. of root (g)	No. of inflorescence/plant
Treatment										
Control	32 ± 1.5^d^	69.67 ± 1.45^d^	7.33 ± 0.33^d^	39.31 ± 0.43^e^	2.62 ± 0.1^d^	0.480 ± 0.025^ed^	9.17 ± 0.4^de^	0.136 ± 0.008^e^	0.082 ± 0.006^f^	7 ± 0,57^cd^
Chilling (6 °C ± 0.5)	27.9 ± 0.49^e^	40.33 ± 1,45^e^	5 ± 0.57^e^	14.28 ± 0.71^f^	2.14 ± 0.24^d^	0.314 ± 0.005 ^e^	9.73 ± 0.2 ^cde^	0.137 ± 0.01^e^	0.0495 ± 0.002^g^	5 ± 0.57^d^
Pot. silicate (80 mM)	36.67 ± 1.2 ^c^	103.33 ± 2.4^a^	9 ± 0.57^bcd^	94.29 ± 0.82^b^	4.83 ± 0.088^b^	0.926 ± 0.04^b^	12.07 ± 1^ab^	0.319 ± −.04^c^	0.114 ± 0.01^d^	11 ± 0.57^ab^
Humic acid (50 mg.l^−1^)	46 ± 1.5^a^	101 ± 0.58^a^	12 ± 0.57^a^	108.84 ± 3.17^a^	7.2 ± 0.65^a^	1.686 ± 0.16^a^	13.37 ± 0.4^a^	0.93 ± 0.088 ^a^	0.245 ± 0.007^a^	12 ± 0.57^a^
γ-ray (50 Gy)	40.17 ± 0.44^b^	89.67 ± 1,45^b^	9.67 ± 0.88^bc^	90.31 ± 1.5^b^	3.87 ± 0.32^bc^	0.8129 ± 0.02^bc^	11.23 ± 0.8^bc^	0.474 ± 0.038^b^	0.197 ± 0.01^b^	8.67 ± 0.88^c^
Chilling + Pot. silicate	34 ± 0.57^cd^	80.33 ± 4.6^c^	8 ± 0.57^cd^	57.39 ± 1.14^d^	2.41 ± 0.06^d^	0.473 ± 0.06 ^de^	10.2 ± 0.15^cd^	0.194 ± 0.006^de^	0.0823 ± 0.003^f^	6.33 ± 0.88^d^
Chilling + Humic acid	36 ± 1.52^c^	93.67 ± 1.8^b^	10 ± 0.57^b^	71.82 ± 1.5^c^	4.1 ± 0.25^b^	0.979 ± 0.04^b^	10.6 ± 0.3^bcd^	0.433 ± 0.02 ^b^	0.138 ± 0.02^c^	9 ± 0.57^bc^
Chilling + γ-ray (50 Gy)	28.33 ± 0.88^e^	80 ± 1.15^c^	8 ± 0.29^cd^	59.34 ± 3.2^d^	3.07 ± 0.37 ^cd^	0.608 ± 0.03^cd^	9.57 ± 0.57^d^	0.285 ± 0.008^cd^	0.108 ± 0.003^e^	8 ± 0.57^cd^
LSD at 0.05	**3.174**	**6.77**	**1.756**	**5.609**	**0.973**	**0.207**	**1.629**	**0.111**	**0.002**	**2.071**
% of inc. by HA over control	**12.5**	**15.6**	**56.5**	**103.9**	**218.4**	**68.3**	**34.4**	**36.4**	**82.7**	**28.6**
% of inc. by HA over chilling-stressed	**29.3**	**8.94**	**91.6**	**208.5**	**216.1**	**178.8**	**132.3**	**100**	**402.9**	**80**

### Yield components

In comparison with non-treated coriander plants, chilling stress (6 °C ± 0.5) induced significant decrease in yield components (c.a. number of fruits/plant, number of seeds/plant, weight of seeds/plant, and weight of 1000 seeds) as shown in Table 4. Among the different treatments, it has been found that, the number of fruits and seeds, seeds weight/plant, and weight of 1000 seeds were all increased. The superior treatment, in enhancing and improving fruits and seeds development within chilling and non-chilling conditions, was HA followed by silicate and γ-radiation. Hereby, HA most likely has triggered the highest ameliorative effect on fruits and seeds number/plant (Table 4). Also, pre-soaking treated coriander seeds in silicate, HA, or γ-radiation have caused improvement of the seed index as compared with control and stressed coriander plants. The best treatment that caused the highest quality and improved seeds yield was HA alone or in combination with chilling. Moreover, seeds quality was improved by 73.3 and 92.92% over those of the control and chilling-stressed plants, respectively (Table 4). Therefore, HA application was the best to alleviate the impact of chilling stress.

**Table 4:** Effect of chilling stress on coriander (*Coriandrum sativum* L) seeds pre- soaked in 80 mM pot. Silicate, 50 mg l^− 1^ humic acid or soaked in water after exposure to γ-rays (50 Gy) and the interaction of the alleviation treatments and chilling stress on the yield components. The shown data was extracted by using 3 biological and 3 technical replicates. Each biological replicate is comprised of 10 plants (one pot). To perform the biochemical analysis, the combined tissue of these ten plants (one pot content) refers to one technical replicate. The readings of the 3 technical replicates were recorded. Sample extraction was done solely for each technical replicate. The mean of the values was used to calculate ±SE. Also, the least significant differences (LSD) at 5% level were calculated to compare the means of different treatments according to Snedecor and Cochran [92]. The values with the same letter are not significantly different (P<0.05). The raw data set of the technical replicates was attached as a supplementary file

Yield component	No. of fruits/plant	No. of seeds/plant	Weight of seeds/plant(g)	Seed index (weight of 1000 seeds (g))
Treatment				
Control	119.0 ± 5.7^g^	238 ± 5.8^g^	0.56 ± 0.08^g^	2.36 ± 0.08^f^
Chilling (6 °C ± 0.5)	77.65 ± 1.14^h^	155.3 ± 1.15^h^	0.32 ± 0.01^h^	2.12 ± 0.012^g^
Pot. silicate (80 mM)	523.50 ± 13.3^b^	1047 ± 13^b^	4.08 ± 0.2^b^	3.9 ± 0.2^b^
Humic acid (50 mg^−1^l)	590.00 ± 5.7^a^	1180 ± 5.6^a^	5.73 ± 0.08^a^	4.86 ± 0.077^a^
γ-rays (50 Gy)	473.65 ± 21.8^c^	947.3 ± 1.15^c^	2.48 ± 0.15^e^	2.62 ± 0.15^e^
Chilling + Pot. silicate	409.65 ± 6.7^e^	819.3 ± 13.3^e^	2.91 ± 0.08^d^	3.56 ± 0.075^c^
Chilling + Humic acid	446.00 ± 48.6^d^	892 ± 13.3^d^	3.65 ± 0.18^c^	4.09 ± 0.19^b^
Chilling + γ-rays	303.00 ± 5^f^	606 ± 5.7^f^	1.81 ± 0.17^f^	2.99 ± 0.16^d^
LSD at 0.05	**27.62**	**27.62**	**0.2072**	**0.2072**

### Endogenous phytohormones

Chilling stress has induced a significant decrease in the growth promoting substances (IAA and GA_3_) levels by 40.68, 42.03%, respectively and significant increase in the growth inhibitor ABA level by 316.6% as compared with control plants (Table 5). All applied treatments either separately or in combination with chilling stress have induced marked increases in both IAA and GA_3_ contents. The maximum increases in IAA (104.52%) and GA_3_ were obtained in chilling-stressed samples alleviated by HA as compared by other chilling-treatments (Table 5). On the other hand, ABA content was increased upon chilling stress and decreased particularly after HA subsequent treatment. Treatment by γ-radiation has led to ABA increase in control coriander. Furthermore, sole treatment by γ-radiation has led to ABA increase in coriander leaves. In addition, chilling stress has caused a marked decrease in IAA+ GA_3_/ABA ratio, while soaking coriander seeds in pot. Silicate, HA, or irradiation with γ-rays has induced a reverse pattern in this ratio as compared with chilling-stressed samples. It was found that the maximum peak of such response was obtained by alleviation of the chilling stress by HA application (Table 5).

**Table 5:** Effect of chilling stress on coriander (*Coriandrum sativum* L*.*) seeds pre- soaked in 80 mM pot. Silicate, 50 mg l^−1^ humic acid or soaked in water after exposure to γ-rays (50 Gy) and the interaction of the alleviation treatments and chilling stress on endogenous phytohormones (μg/100 F. wt.) at flowering stage. The shown data was extracted by using 3 biological and 3 technical replicates. Each biological replicate comprised of 10 plants (one pot). To perform the biochemical analysis, the combined tissue of these ten plants refers to one technical replicate. The readings of the 3 technical replicates were recorded. Sample extraction was done solely for each technical replicate. The mean of the values was used to calculate ±SE. Also, the least significant differences (LSD) at 5% level were calculated to compare the means of different treatments according to Snedecor and Cochran [92]. The values with the same letter are not significantly different (P<0.05). The raw data set of the technical replicates was attached as a supplementary file

phytohormone	IAA	GA_3_	ABA	IAA + GA_3_/ABA
Treatment				
Control	7.08 ± 0.05^e^	390.00 ± 17.3^e^	1.75 ± 0.43^h^	226.51
Chilling (6 °C ± 0.5)	4.20 ± 0.12^f^	226.10 ± 17.3^h^	7.29 ± 0.43^b^	31.61
Pot. silicate (80 mM)	13.20 ± 0.69^b^	405.51 ± 17.3^c^	3.90 ± 0.43^e^	107.4
Humic acid (50 mg l^−1^)	15.54 ± 0.69^a^	443.43 ± 17.3^a^	4.07 ± 0.43^d^	112.7
γ- rays (50 Gy)	10.86 ± 0.69^d^	390.86 ± 17.3^d^	7.74 ± 0.43^a^	51.9
Chilling + Pot. silicate	6.24 ± 0.69^e^	326.93 ± 17.3^f^	3.10 ± 0.43^f^	107.27
Chilling + Humic acid	8.59 ± 0.69^d^	423.45 ± 17.3^b^	2.44 ± 0.43^g^	177.1
Chilling + γ- rays	6.86 ± 0.69^e^	255.23 ± 17.3^g^	4.38 ± 0.43^c^	59.83
LSD at 0.05	**0.862**	**24.49**	**0.613**	**–**

### Changes in photosynthetic pigments and carbohydrates content

Chilling stress caused a pronounced decrease in chl a, chl b, and consequently the total chlorophylls below those detected in control coriander leaves. All applied treatments have induced a marked increase in chl a, chl b, and total chlorophylls in stressed samples (Table 6). The maximum alleviated impact was achieved by individual treatment of HA or HA combined with chilling when compared with chilling-stressed coriander samples. Chilling stress has induced an increase in (chl *a*/chl *b*) ratio more than control plants. Furthermore, all applied treatments have triggered a marked increase in (chl *a*/chl *b*) ratio in relation to control. Chilling stress combined with different stimulator elements (Pot. silicate, HA, and γ-irradiation) have recorded an increase in (chl *a*/chl *b*) ratio in control and chilling stress leaves. The maximum values 2.14 were achieved by chilling plus Pot. silicate and chilling plus HA which increased by 20.90 and 12.04% more than control and chilling-stressed leaves, respectively. On the other hand, the soluble sugars were increased significantly in chilling stressed plants, particularly under the effect of HA treatment compared with control values. All applied treatments- Pot. silicate, HA, or gamma radiation- either separately or in combination with chilling stress have increased the soluble sugars content of coriander leaves as compared with untreated control plant (Table 6). The most pronounced effect was recorded in HA application. The latter treatment was considered as the best enhancer for soluble sugars in chilling stressed coriander by 40% higher than control samples, followed by gamma radiation and Pot. silicate application. Polysaccharide contents were decreased under chilling stress and increased in treated coriander alleviated with silicate and HA in both stressed and control coriander. However, the increase of total carbohydrates level was taken place by HA pretreatment in the control and alleviated chilling stressed coriander with silicate and HA (Table 6). It was worthy to mention that although individual gamma radiation has increased carbohydrates values over chilling stress condition, it was not the best in terms of chilling stress alleviation through carbohydrates protection and restoration compared to HA and silicate treatments (Table 6). Gamma rays’ impact on carbohydrates might be described as intermediate between HA and pot. Silicate effects.

**Table 6:** Effect of chilling stress on coriander (*Coriandrum sativum* L*.*) seeds pre- soaked in 80 mM pot. Silicate, 50 mg l^−1^ humic acid or soaked in water after exposure to γ-rays (50 Gy) and the interaction of the alleviation treatments and chilling stress on photosynthetic pigments (μg/g D. wt. in coriander leaves) and carbohydrate contents (g/100 g D. wt.) at flowering stage. The shown data was extracted by using 3 biological and 3 technical replicates. Each biological replicate comprised of 10 plants (one pot). To perform the biochemical analysis, the combined tissue of these ten plants refers to one technical replicate. Sample extraction was done solely for each technical replicate. The readings of the 3 technical replicates were recorded. The mean of the values was used to calculate ±SE. Also, the least significant differences (LSD) at 5% level were calculated to compare the means of different treatments according to Snedecor and Cochran [92]. The values with the same letter are not significantly different (P<0.05). The raw data set of the technical replicates was attached as a supplementary file

Pigment/Carbohydrate	Photosynthetic pigments				Carbohydrate fractions		
Treatment	Chl a	Chl b	Chl a/b	Total Chl	Soluble Sugars	Polysaccharides	Total Carbohydrates
Control	13.12 ± 0.01^e^	7.43 ± 0.13^b^	1.77 ± 0.03	20.55 ± 0.15^bc^	2.26 ± 0.05^e^	14.39 ± 0.88^c^	16.65 ± 0.9^cd^
Chilling (6 °C ± 0.5)	9.72 ± 0.16^g^	5.1 ± 0.2^d^	1.91 ± 0.11	14.82 ± 0.05^d^	2.9 ± 0.049^cd^	9.46 ± 0.36^e^	12.36 ± 0.3^e^
Pot. silicate (80 mM)	15.12 ± 0.35^b^	7.9 ± 0.005^b^	1.91 ± 0.04	23.02 ± 0.36^b^	3.59 ± 0.11^ab^	19.17 ± 1.07^a^	22.76 ± 1.1^a^
Humic acid (50 mg l^−1^)	17.50 ± 0.18^a^	9.53 ± 0.02^a^	1.84 ± 0.01	27.03 ± 0.12^a^	4.06 ± 0.06^a^	21.57 ± 0.34^a^	25.16 ± 0.3^a^
γ- rays (50 Gy)	14.86 ± 0.21^bc^	7.91 ± 0.12^b^	1.88 ± 0.05	22.77 ± 0.18^b^	2.49 ± 0.19^de^	16.32 ± 0.5^b^	18.81 ± 0.3^b^
Chilling + Pot. silicate	14.14 ± 0.36 ^d^	6.61 ± 0.24^c^	2.14 ± 0.02	20.75 ± 0.6^bc^	2.55 ± 0.08^de^	15.43 ± 0.59^bc^	17.97 ± 0.5^bc^
Chilling + Humic acid	14.17 ± 0.12^cd^	6.61 ± 0.05^c^	2.14 ± 0.002	20.78 ± 0.17^bc^	3.35 ± 0.32^bc^	15.69 ± 0.6^bc^	19.04 ± 0.84^b^
Chilling + γ- rays	12.30 ± 0.08^f^	5.85 ± 0.02^d^	2.10 ± 0.12	18.15 ± 0.09^c^	3.08 ± 0.06^cd^	11.87 ± 0.22^d^	14.95.4 ± 0.28^d^
LSD at 0.05	**0.72**	**0.59**	**0.067**	**1.55**	**0.418**	**1.966**	**0.006**

### Changes in antioxidant compounds

The Changes in antioxidant compounds (ascorbic acid, carotenoids, flavonoids, total Phenols, and Proline) of coriander leaves in response to pre-sowing step with chilling stress (the case of pot. Silicate or HA) or in H_2_O (after exposed to γ- radiation) and their interaction were shown in Table 7.

**Table 7:** Effect of chilling stress on coriander (*Coriandrum sativum* L*.*) seeds pre-soaked in 80 mM pot. Silicate, 50 mg l^− 1^ humic acid or soaked in water after exposure to γ-rays (50 Gy) and the interaction of the alleviation treatments and chilling stress on antioxidant compounds (ascorbic acid, carotenoids, flavonoids, total phenolics and proline) at flowering stage. The shown data was extracted by using 3 biological and 3 technical replicates. Each biological replicate comprised of 10 plants (one pot). To perform the biochemical analysis, the combined tissue of these ten plants refers to one technical replicate. Sample extraction was done solely for each technical replicate. The readings of the 3 technical replicates were recorded. The mean of the values was used to calculate ±SE. Also, the least significant differences (LSD) at 5% level were calculated to compare the means of different treatments according to Snedecor and Cochran [92]. The values with the same letter are not significantly different (P<0.05). The raw data set of the technical replicates was attached as a supplementary file. The percentage of increase (inc.) or decrease (dec.) caused by the chilling stress was investigated. ⬇ Refers to the percentage of decrease and ⬆ refers to the percentage of increase compared with the control values. By being the best alleviation element against the chilling stress (except for phenolic and proline), the percentage of increase in all measurements, triggered by HA application, was further investigated. This percentage was calculated by subtracting the value of control/chillied reading from the reading value of any physiological treatment, then the result was divided by the reading of control value, and finally, the result is multiplied by 100

Antioxidant compounds	Ascorbic acid (g/100 g) D. wt.	Carotenoids (μg/g) D. wt.	Flavonoids (g/100 g) D. wt.	Phenolic (g/100 g) D. wt.	Proline (μg/100 g) D. wt.
Treatment					
Control	0.47 ± 0.03^d^	4.63 ± 0.24^b^	0.476 ± 0.04^h^	0.968 ± 0.021^c^	166.3 ± 2.88^c^
Chilling (6 °C ± 0.5)	0.25 ± 0.01^h^	2.54 ± 0.16^c^	0.571 ± 0.043^d^	1.14 ± 0.021^c^	320.6 ± 2.88^b^
Pot. silicate (80 mM)	0.54 ± 0.01^c^	6.04 ± 0.26^a^	0.563 ± 0.04^e^	1.13 ± 0.021^d^	242.4 ± 2.88^d^
Humic acid (50 mg l^−1^)	0.73 ± 0.11^a^	6.07 ± 0.11^a^	0.686 ± 0.04^a^	1.37 ± 0.021^a^	191.1 ± 2.88^f^
γ - rays (50 Gy)	0.44 ± 0.18^f^	5.80 ± 0.15^a^	0.498 ± 0.043^f^	0.995 ± 0.015^e^	228.6 ± 2.88^e^
Chilling + Pot. silicate	0.48 ± 0.01^d^	5.65 ± 0.11^a^	0.59 ± 0.043^c^	1.179 ± 0.045^b^	326.4 ± 2.88^a^
Chilling + Humic acid	0.62 ± 0.01^b^	5.89 ± 0.05^a^	0.659 ± 0.04^b^	0.988 ± 0.021^f^	277.6 ± 2.88^c^
Chilling + γ – rays	0.37 ± 0.01^g^	4.58 ± 0.12^ab^	0.473 ± 0.04^h^	0.950 ± 0.021^g^	173.0 ± 2.88^g^
LSD at 0.05	**0.0017**	**0.277**	**0.00175**	**0.00175**	**0.0017**
% of inc./dec. caused by chilling stress compared with control	⬇**46.81**	⬇**48.79**	⬆**19.96**	⬆**46.9**	⬆**93.95**
% of inc. caused by HA compared with chilling stress	**148**	**131.89**	**15.41**	**–**	**–**

Firstly, referred to control value, chilling stress caused a significant decrease in ascorbic acid contents by 46.81% below control value. In turn, pot. Silicate and HA (separate or in combination with chilling stress) have caused a significant increase in ascorbic acid contents as compared with non-chilling & chilling-stressed plant. The maximum value obtained from chilled plant primed in HA was increased reached 31.91 and 148% over non-chilling and chilling control plant, respectively (Table 7). Conversely, exposure of seeds to γ- rays caused a decrease in ascorbic acid contents as compared with control plant, but their interaction with chilling stress have induced marked increase in ascorbic acid as compared with chilling-stressed plant. The most effective treatment in alleviating adverse effect of chilling was HA.

Secondly, it was notably detected that chilling in coriander caused a significant decrease in carotenoids content by 45.14% below of control plants. Pot. silicate, HA, and γ- radiation and their interaction with chilling stress have induced marked increments in carotenoids content over chilling-stressed plants. The most effective treatment in alleviating adverse effect of chilling was HA followed by silicate individually or in combination with chilling stress (Table 7).

Thirdly, as compared with the control coriander plants, chilling stress has caused a significant increase in flavonoids content by 19.96% as shown in Table 7. While, silicate, HA, and γ- radiation individually caused significant increments in flavonoids content in relation to non-chilling control. The highest content was obtained by seeds soaked in HA. However, the interaction between different treatments and chilling stress induced significant increments in flavonoid contents as compared with non-chilling or chilling stressed control plants except in γ- radiation, which decreased flavonoids content significantly when compared to chilled control plants. Therefore, the best treatment that alleviated the harmful effect of chilling was HA then silicate, as both had increased the flavonoids content by 15.41 and 3.33% over that of chilled coriander plants.

Fourthly, compared with control plants, chilling stress induced increments in total phenolic content by 17.768% over control coriander plant. Silicate, HA, and γ- radiation which applied individually and their interactions with chilling stress induced significant increments in total phenol contents as compared with non-chilling control plants. On the other hand, the interaction treatments decreased total phenol except silicate that caused an increase by 3.42% as compared with chilled plants.

Finally, incubation of coriander seed in water (6 °C ± 0.5) for 16 h has increased proline contents by 92.78% in grown leaves above the control value. Generally, silicate, HA, and gamma irradiation treatments have induced increments in proline content comparing to non-chilling control plants. However, all applied treatments decreased the proline contents below those of chilling stressed plant except in case of silicate in combination with chilling stress, which increased their content by 1.81%.

### Antioxidant enzymes and lipid peroxidation

The changes in antioxidants enzymes activities were investigated for primed non-chilled or primed chilled coriander plant using pot. Silicate, HA, and H_2_O after exposure to gamma rays and their interaction are represented in Table 8. All the applied treatments have decreased PPO activity below that of chilling stressed plant, except in plant exposed to γ- radiation, which has non-significant change. Also, alleviation the chilling stress by pot. Silicate, HA, and γ-radiation decreased POD activity by 33.46, 38.62, and 53.35%, respectively below the primed chilled plant but its values were still markedly higher than those of control plants by 133.56, 115.44, and 63.76%, respectively (Table 8). Conversely, CAT activity in leaves of soaked coriander in HA, or exposed to gamma radiation decreased by 30.79 and 64.90% as compared with non-chilled plants and by 66.29 and 82.9% compared to chilled plants. In addition, the interaction between chilled plants and all applied treatments caused a decrease in CAT activity below the control and chilled plant except in silicate and silicate combined with chilled plant, which caused an increase by 57.62 and 14.57% compared with non-chilled control plants, respectively.

**Table 8:** Effect of chilling stress on coriander (*Coriandrum sativum L.*) seeds pre- soaked in 80 mM pot. Silicate, 50 mg l^−1^ humic acid or soaked in water after exposure to γ-rays (50 Gy) and the interaction of the alleviation elements and chilling stress on antioxidant enzymes PPO, POD, CAT (unit/mg protein) and MDA(nmol/g F. wt.) at flowering stage. The shown data was extracted by using 3 biological and 3 technical replicates. Each biological replicate comprised of 10 plants (one pot). To perform the biochemical analysis, the combined tissue of these ten plants refers to one technical replicate. Sample extraction was done solely for each technical replicate. The readings of the 3 technical replicates were recorded. The mean of the values was used to calculate ±SE. Also, the least significant differences (LSD) at 5% level were calculated to compare the means of different treatments according to Snedecor and Cochran [92]. The values with the same letter are not significantly different (P<0.05). The raw data set of the technical replicates was attached as a supplementary file. The percentage of increase (inc.) caused by the chilling stress compared with the control values was investigated. By being the most alleviation element to restore quietly the enzymes steady state concentration (except for POD and MDA), the percentage of decrease compared with the chilling stress values in all measurements triggered by HA application was further investigated. This percentage was calculated by subtracting the value of control/chillied reading from the reading value of any physiological treatment, then the result was divided by the reading of control value, and finally, the result is multiplied by 100

Antioxidant enzyme/MDA	Antioxidants enzyme (unit/mg protein)			Lipid peroxidation (nmol/g F. wt.)
Treatment				
	PPO	POD	CAT	MDA
Control	5589.2 ± 115^cd^	1.49 ± 0.12^e^	0.604 ± 0.06^c^	0.481 ± 0.01^e^
Chilling (6 °C ± 0.5)	8966.5 ± 577^a^	5.23 ± 0.12^a^	1.24 ± 0.1^a^	0.888 ± 0.03^a^
Pot. Silicate (80 mM)	6821.5 ± 58.9^b^	3.08 ± 0.12^bcd^	0.952 ± 0.19^b^	0.582 ± 0.02^cd^
Humic acid (50 mg l^−1^)	4608.5 ± 86^ef^	3.88 ± 0.42^b^	0.418 ± 0.1^cd^	0.642 ± 0.02^bc^
γ-rays (50 Gy)	4059.4 ± 209^f^	2.95 ± 0.12^cd^	0.212 ± 0.002^d^	0.558 ± 0.019^de^
Chilling + Pot. silicate	6226.6 ± 101^bc^	3.48 ± 0.69^bc^	0.692 ± 0.1^bc^	0.703 ± 0.027^b^
Chilling + Humic acid	4996.4 ± 288^de^	3.21 ± 0.12^bcd^	0.596 ± 0.02^c^	0.702 ± 0.024 ^b^
Chilling + γ-rays	8234.01 ± 126^a^	2.44 ± 0.43^d^	0.212 ± 0.002^d^	0.572 ± 0.026^cd^
LSD at 0.05	**758**	**0.8964**	**0.2769**	**0.078**
% of inc. in chilling values compared with control	**60.4**	**251**	**106**	**84.61**
% of dec. in Chilling + HA compared chilled plants	**44.3**	**38.6**	**51.93**	**20.94**

Regarding monitoring lipid peroxidation, estimation of MDA is crucial since MDA was a marker for evaluating lipid peroxidation and damage to plasma lemma or organelle membranes which increases with different environmental stress factors. The result listed in Table 8 revealed that incubation of coriander plant seeds in 6 °C ± 0.5 induced a marked increase in MDA contents by 84.62% with respect to control coriander plant. Whereas pre-soaked seeds in pot. Silicate or HA or soaking in water after irradiated by γ- rays have induced either significant increase in MDA values as compared with control coriander plants or decrease by 34.45, 27.70 and 37.16%, respectively when compared with chilling stress. Also, the interaction of priming and chilling caused a decrease in value of MDA in relation to chilling stressed plants. The magnitude of such response was more pronounced in gamma radiation followed by HA priming, which decreased by 35.59 and 20.95%, respectively. In general, pot. Silicate, HA, and γ- radiation could alleviate the inhibitory effect of chilling stress by decreasing lipid peroxidation below that induced by chilling stress.

### Characterization of chilling stress impact on TCPs and expression of chloroplast marker proteins

**Fig. 1:**
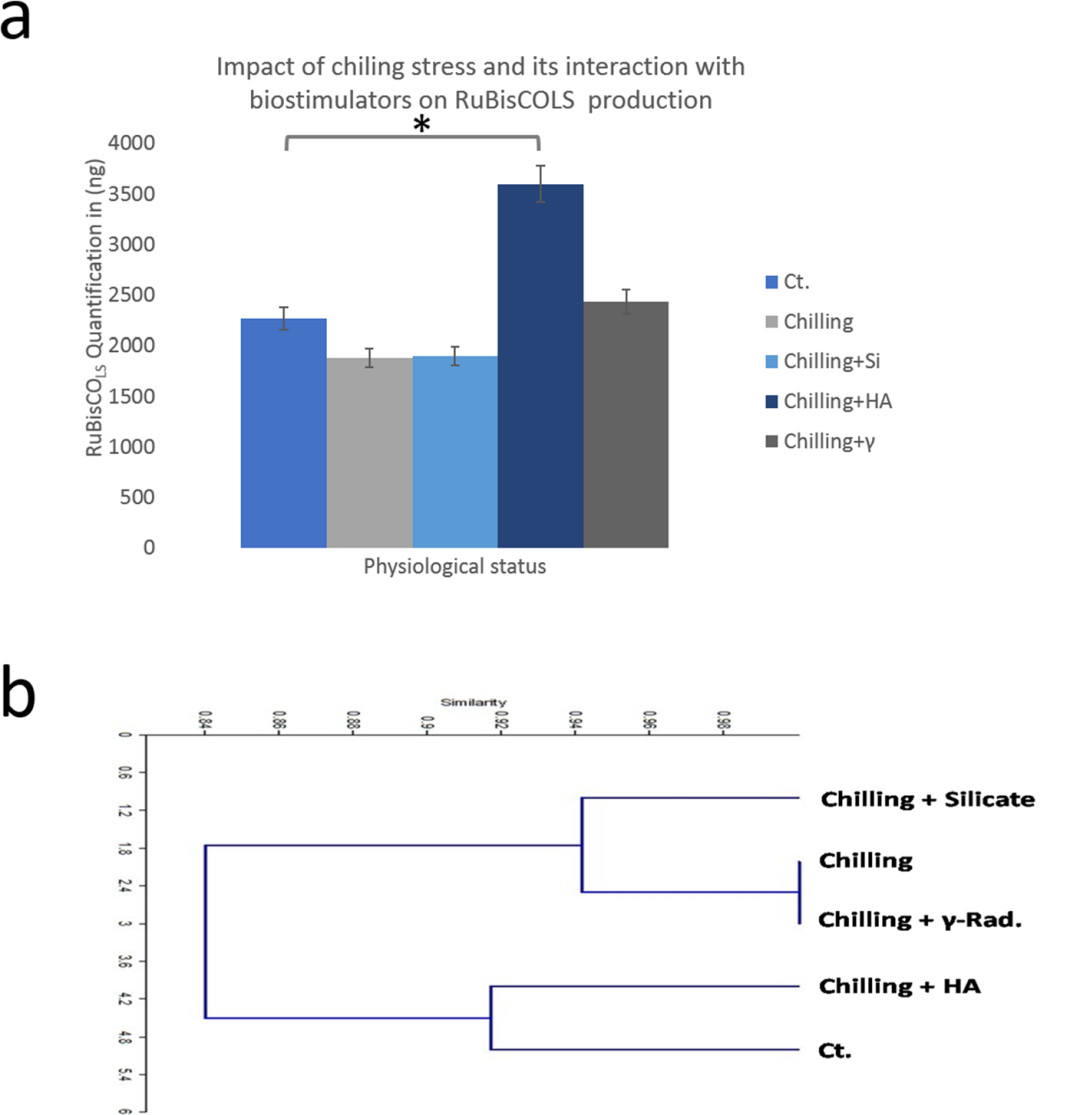
**a)** Changes in the steady state expression level of RuBisCO_LS_ protein**.** Y axis values indicates the normalized protein production of differentially expressed RuBisCO_LS_ protein in nanograms (ng) of three independent biological replicates and three technical ones. The data were shown as mean ± s.e.m.; *, *P* < 0.05. ImageJ software (IJ 1.46r) was used for image processing and analysis of the electrophoretic running of ascending concentration series of BSA (as protein size standard) to quantify RuBisCO_LS_ concentration in (ng) of three independent gel repeats. Full data sets showing the quantification counts were supplement separately. Also, Full-length gels are presented in Supplementary Fig. (2a and b). The data was normalized to the protein band running approximately at 180 kDa as shown in Suppl. Fig. 2a. **b)** Impact of alleviation treatments on TCPs profiles of chilling-stressed (6 °C ± 0.5) coriander plants at the vegetative stage (75 days old). Cluster analysis resulted from SDS-PAGE fractionated TCPs as revealed by chilling stress and alleviation treatments to its impact. A dendrogram for the five examined coriander samples was constructed using scored data of fractionated TCPs after chilling stress application and subsequent biostimulants treatments using **U**nweighed **P**air-**g**roup **M**ethod of **A**rithmetic mean (UPGMA) and similarity matrices was computed according to Dice coefficient

**Fig. 2:**
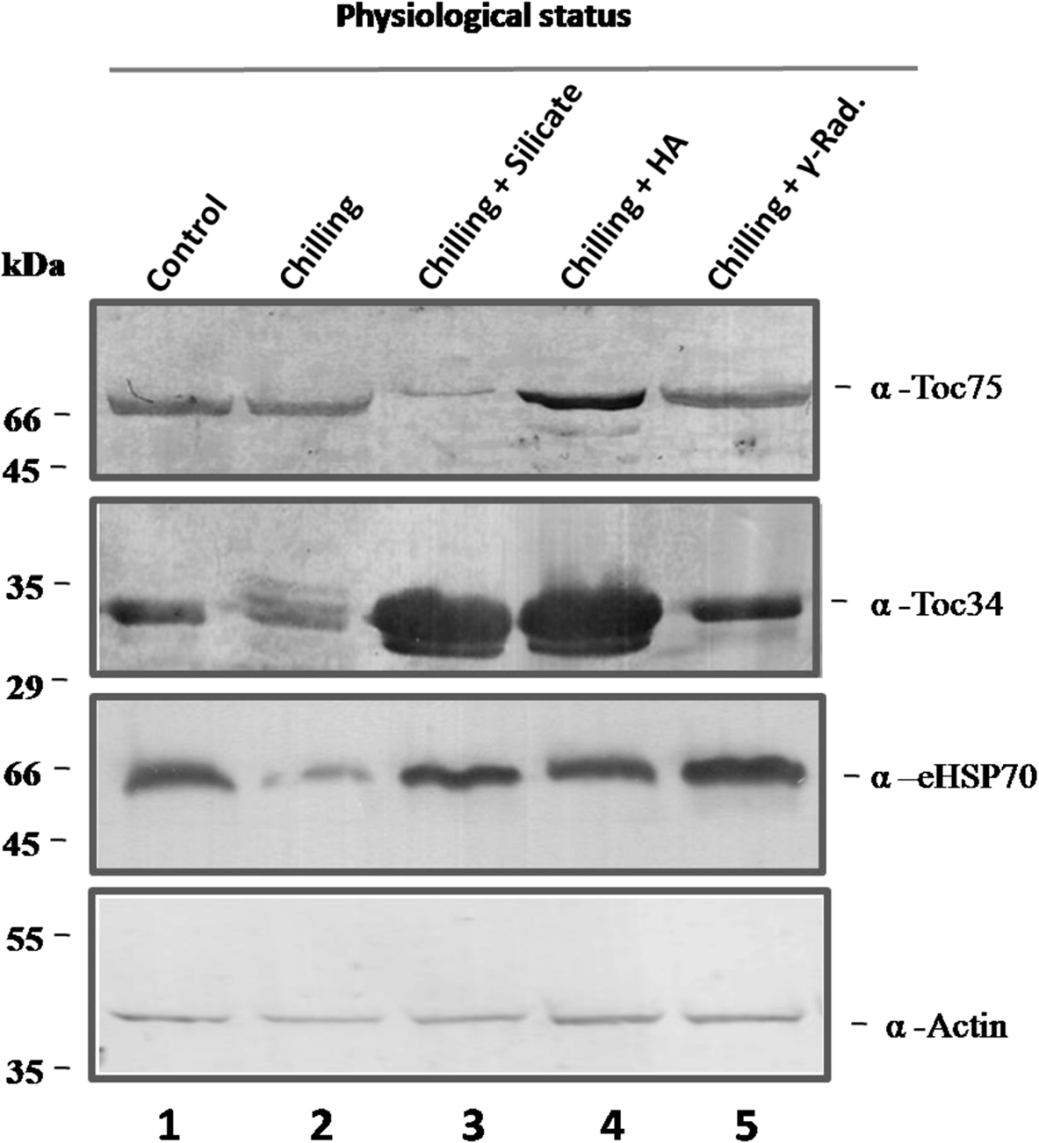
Immunoblot analysis of the expression of chloroplast marker proteins. Coriander control (Lane 1) and chilling-stressed (Lane 2) ones were subjected to combination of chilling stress in presence of 80 mM Pot. silicate (Lane 3), 50 mg l^− 1^ HA (Lane 4) or soaked in water after exposed to 50 Gy gamma irradiation (Lane 5). TCPs were extracted and fractionated by SDS-PAGE and immunodecorated against α-Toc34, α-Toc75, eHSP70, and actin primary antibody in a dilution of 1:10,000 as demonstrated in [51]. Cropping of the shown blots was performed properly for sake of clarity and focusing the information. Full-length blots are accompanied the manuscript as Supplementary Fig. 3. Protein extraction procedure for each physiological status (control, chilling stressed, etc.) was performed from the leaves of 3 biological replicates and 3 technical replicates. Each technical replicate represented one biological replicate. Each biological replicate comprises the collection of leaves of 10 plants. The protein extraction was carried out from each technical replicate independently. Finally extracted proteins from the 3 technical replicates were pooled together. Pooled sample were quantified, equally loaded into 10% SDS-PAGE, and blotted onto PVDF membrane as shown in methods section. Consequently, aliquots of pooled sample were kept as −80 °C after the short snap for 30 s in Liquid Nitrogen

**Fig. 3:**
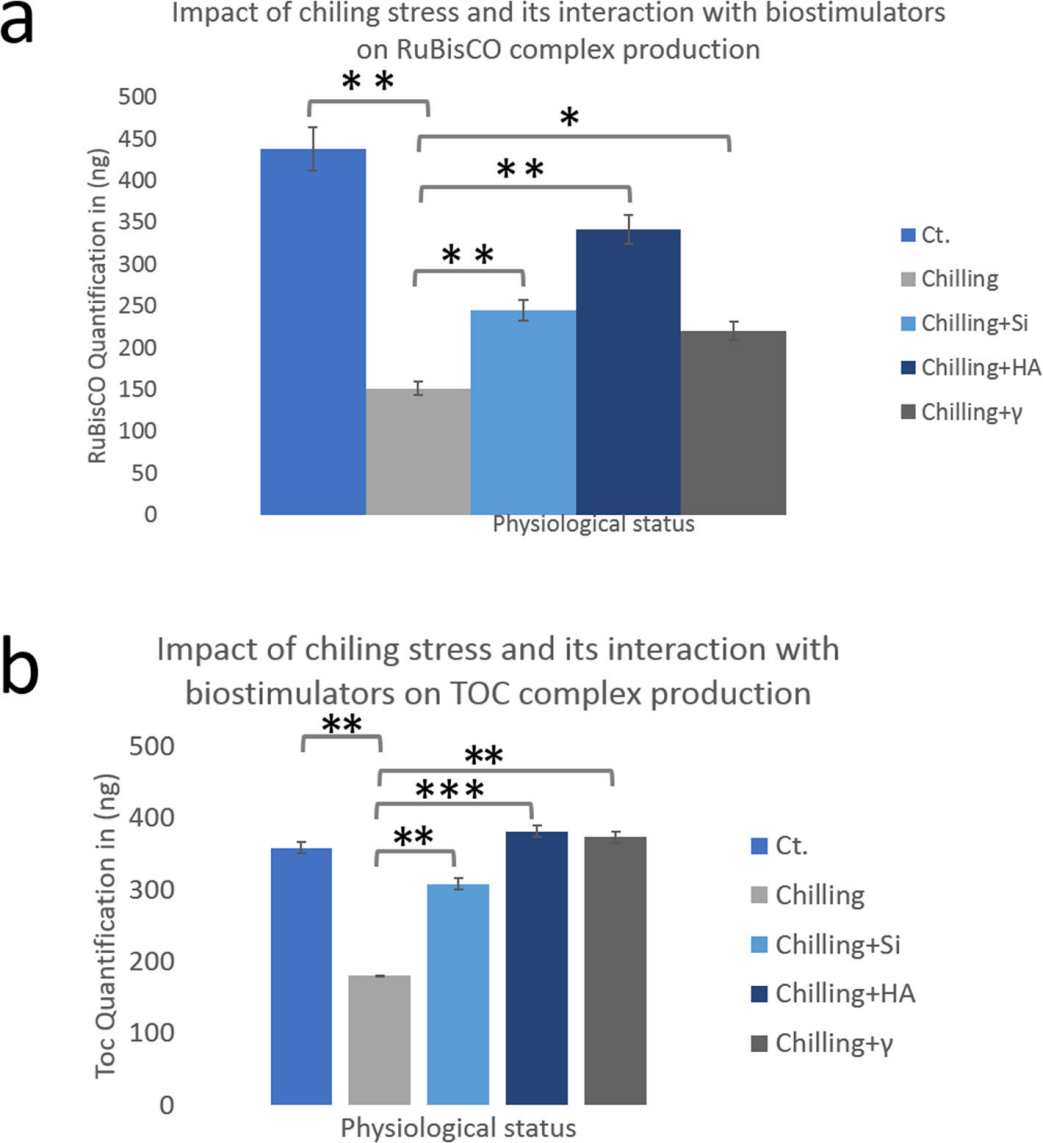
Changes in the steady state expression level of RuBisCO (panel a) and Toc (panel b) protein complexes**.** Y axis values indicates the normalized protein production of differentially expressed RuBisCO/Toc complexes in nanograms (ng) of three independent biological experiments and three technical ones. The data were shown as mean ± s.e.m.; *, *P* < 0.05, **, *P* < 0.005, ***, and *P* < 0.0005. ImageJ software (IJ 1.46r) was used for image processing and analysis of the electrophoretic running of ascending concentration series of BSA (as protein size standard) to quantify the concentration in (ng) of three independent gel repeats. Full data sets showing the quantification counts were supplement separately. Also, Full-length gels are presented in Supplementary Fig. (3a, b). The data was normalized to the protein band running approximately at 300 kDa as shown in Suppl. Fig. 3a

TCPs were extracted from control, chilling-stressed, and alleviated biostimulants treated and stressed leaves of 75-days-old coriander plant at vegetative stage. Protein banding profiles of 70–100 μg TCPs (equivalent to total protein content) were fractionated using 10% SDS-PAGE technique (Fig. S2a, b). To manifest the consistency and reproducibility of resulted protein profiles after stress performing and stress-alleviation application, TCPs were extracted from studied samples along with two successive seasons (season 1; Fig. S2a and season 2; Fig. S2b). It was found that the protein band, detected approximately at 53 kDa, was identified as RuBisCO_LS_ in all samples of control (Fig. S2a, b, Lane 1), stressed (Fig. S2a, b, Lane 2), and chilling-stressed alleviated coriander plants (Fig. S2a, b, Lanes 3–5). Accumulation of RuBisCO_LS_ was pronounced and negatively affected by applied chilling stress (Fig. 2a). Notably, alleviation of chilling stress by HA application (50 mgl^− 1^) has potentially enhanced and promoted the accumulation of the major pronounced RuBisCO_LS_ protein band **(**Fig. S2a, b). The Expression of RuBisCO_LS_ protein product was not retrieved, at least to control level, in chilling-stressed coriander plants alleviated by separate and individual application of silicate and gamma irradiation **(**Fig. S2a, b). Moreover, using of HA as a stress alleviation element has positively induced the expression of unique and characteristic polypeptides running approximately at 45, 48, 65, and 80 kDa more than their corresponding bands in control samples (Fig. S2a, b). In the same context, quantification of RuBisCO_LS_ protein band, by loading ascending concentrations of protein standard BSA using SDS-PAGE technique, has manifested previous investigations (Fig. 1a; Fig. S2a, b). Band scoring has revealed a percentage of polymorphism by 38.4 and 29.4% for season 1 and 2, respectively, with a mean of 33.9%. The generated binary matrix (based on band presence) was used to construct a cluster analysis. Latter analysis was used to find the most relevant samples based on their protein profiles. Notably, control coriander was clustered in one group with stressed samples alleviated by HA treatment (Fig. 1b). On the other hand, the expression of Toc34, Toc75, and eHSP70 were negatively affected by chilling stress; whereas HA treatment was able to maintain, even up-regulate, their production (Fig. 2; Fig. S3). The same findings were demonstrated concerning the expression of RubisCO and Toc complexes running approximately at 480 and 700 kDa, respectively (Fig. 3; Fig. S4). HA was found to trigger the optimum alleviating impact keeping and promoting the production of both RubisCO and Toc complexes (Fig. 3).

## Discussion

### Improvement of the growth parameters and yield components in stressed-alleviated coriander plants

Generally, all growth parameters were stimulated by soaking seed in potassium silicate, HA or exposed to γ-rays as compared with control and chilling (stressed) coriander plants (Table 2). The most effective treatment was HA alone in both control and chilling- primed samples. In the present study, chilling stress has initiated adversely and inhibitory impact on investigated growth parameters in coriander plant. Reduction in shoot length, branches number/plant, leaves area/plant, root length, fresh and dry weights of shoot and root at vegetation and flowering stages of plant was observed. As compared with control values, this reduction in growth parameters has been elsewhere reported [29, 32] and could be attributed to decrease in water absorption, altered cell division and cell elongation rates which affect the leaf sizes and weight and reduced ability to close stomata in response to subsequent water deficit [17]. Supply of insufficient water provoked a rapid drop of water potential in leaves during the first hours of cooling. The declining rate of photosynthesis, due to the adverse effect in CO_2_ assimilation, may weaken the growth through lowering of the rates of both cell division and elongation [4]. Improvement in the growth parameters by increasing of shoot length, fresh and dry weight of shoot and root, leaf area, and branches number/plant (Table 2) were initiated and triggered by using silicate, HA, and gamma rays to alleviate chilling stress. The most effective alleviating element was HA in both control and chilling-stressed samples. The triggered stimulatory impact in growth parameters could be considered as a protective role of silicate, HA, and gamma rays. Silicon was suggested to alleviate chilling stress by deposition in cell wall, increasing its rigidity, and increasing internal storage water within the plant by reducing the water loss, conferring higher growth rates, and, lightening in turn harmful effects of abiotic stress [10]. Also, application of HA was suggested to induce plant growth by acting as a plant growth regulator [80] by the interaction of HA with the rhizosphere and evolving IAA increasing cell division. The latter promotional results were reflected as an increase in cytoskeleton protein, growth of lateral roots, and root total area [19, 73]. Detected IAA higher rate in coriander treated plants with HA supported the latter notion. HA might lead to higher rates of K+ ions uptake and therefore a corresponding increase in chlorophyll fluorescence [67]. Hereby, it might be suggested that HA has improved plant tolerance to abiotic stress and promoted growth by increasing auxins, gibberellins and decreasing ABA (the present data), enhancing nutrient uptakes, photosynthesis, and by reduction of water loss [21, 84]. In addition, stimulation effect of low doses of gamma rays was evidenced by the promotion of various cellular processes, induction the biosynthesis of phytohormones or nucleic acid, accelerated cell proliferation and enzymatic activity, stress resistance, and crop yield [48, 78].

The results obviously have shown that pre-sowing coriander seeds in HA was the most effective treatment in mitigation the adverse effect of chilling on seeds yield of coriander plant (Table 4). This result agreed with an earlier study [11]. Improvement of yield and yield components by HA may be attributed to increasing of nutrients uptake, especially nitrogen content, phosphorus and hormone-like effect of HA, or by maintained photosynthetic tissues and leaf chlorophyll increase [74]. Also, the stimulatory effect of endogenous hormones on the cell division and/or enlargement by applied HA was reported by maintaining IAA level, decreasing IAA oxidase activity, and promoting metabolic activities which accelerate crops growth and yield [42]. In addition, gamma irradiation has induced improvement of seed yield in the chilling of coriander plants. Similar results were obtained for sunflower [2], *Ammi visnage* L. **[**24**]**, and soybean [72]. This could be ascribed to growth stimulation by changing the hormonal signaling network, or by increasing antioxidative capacity of the cell to easily overcome daily stress [47], or by promoting the enzymatic activation resulting in stimulation of cell division rate, which affects not only in germination but also vegetative growth and flowering. In the same context, previous studies have concluded that plant and grain nutritional quality were enhanced by irradiation due to its promoting effect on plant water status by controlling photosynthetic rate, transpiration, and stomatal conductance [90].

### HA is the key player in promotion of endogenous phytohormones under chilling stress

Chilling stress caused a decrease in both IAA and GA_3_ contents in coriander leaves (Table 5). This may be due to the influence of chilling stress on hormonal balance that affects plant growth and development. Hereby, it could be speculated that the reduction in plant growth under stress conditions could be an outcome of an altered hormonal balance [70]. On the other hand, the amount of ABA detected in coriander leaves increased in response to chilling stress. Abscisic acid accumulated in response to different environmental stresses such as salinity, cold and drought [39]. ABA regulates important cellular processes such as stomatal closure by guard cells, mediated by solute efflux, and regulates expression of many genes that may function in tolerance against chilling stress [39]**.** On the other hand, pre-soaking coriander seeds in silicate, HA, and irradiation with gamma rays induced higher contents of growth promoting substances (IAA and GA_3_) and lowered ABA level. The most effective treatment that increased (IAA and GA_3_) to alleviate chilling stress was HA (Table 5). In this respect, latter findings agreed with Abdel-Mawgoud et al. [1] who has demonstrated that HA treatment was the causal agent of increased auxins, cytokinins, and GA_3_ contents in tomato. In the same context, growth promoter (IAA) increased in wheat grown under newly reclaimed soil supplemented with HA [23]. HA might be considered as growth regulator that adjusts hormonal levels, stimulates plant growth, and induces stress tolerance [21]. To a lesser extent, low dose of γ-rays was found to increase Kinetin and GA_3_ hormones of *Eruca vesicaria* L. through triggering changes in hormonal signal network followed by stimulation of growth [71]. It might be concluded that improvement of coriander tolerance to chilling stress was achieved to a higher extent in response to applied HA treatment, followed by silicate. This depended on their role in decreasing IAA oxidase activity, synthesizing adequate level of endogenous phytohormones, promoting metabolic activity, and consequently accelerating plant growth.

### Enhancement of photosynthetic pigments by HA in coriander plants with alleviated stress

The deleterious effect of chilling stress on photosynthetic pigments of coriander leaves was shown through decreasing chl a, chl b, and subsequently the total chlorophylls (Table 6). This result was consistent with earlier experiments conducted on *Phaseolus* spp. grown at low temperature (10 °C) [97]. The marked reduction in photosynthetic pigments in chilling-stressed coriander leaves might be ascribed to the mechanical forces generated by formation of extracellular ice crystals, cellular dehydration, and increase concentration of intracellular salts [55]. Latter mechanical forces not only resulted in membrane damage and membrane structure alteration but also affected photosynthetic electron transport, CO_2_ fixation, RubisCO activity, and stomatal conductance [61, 62]. Application of silicate, HA, and gamma radiation on chilling-stressed plants could alleviate the adverse effect of chilling by increasing Chl a, Chl b, and the total chlorophylls levels (Table 5). These results were in harmony with those of Zhu et al. [103], Sivanesan et al. [91], and Habibi [29]. This may be attributed to silicon whose application increased the levels of chl a and chl b, which in turn indicates synthesis of new pigments and maintenance of previously existing chl a and chl b. However, HA was the most effective treatment in mitigating chilling stress by increasing Chl a, Chl b, and consequently total chlorophylls. This may be ascribed to the role of HA as an important biostimulant capable of promoting hormonal activity, producing antioxidants, and reducing free radicals in plants. It has improved root vitality, increased nutrient uptake, stimulated chlorophyll synthesis and/or delayed chlorophyll degradation [57]. Taken together from the presented results, HA treatment restored and maintained the hormonal balance in chilling-stressed coriander to the same level found in the control plants. This balance was triggered by declining ABA levels which mediated root growth enhancement, maintained photosynthetic pigments, and carbohydrates metabolism [63, 70].

### Enrichment of carbohydrate content by HA treatment

**Fig. 4:**
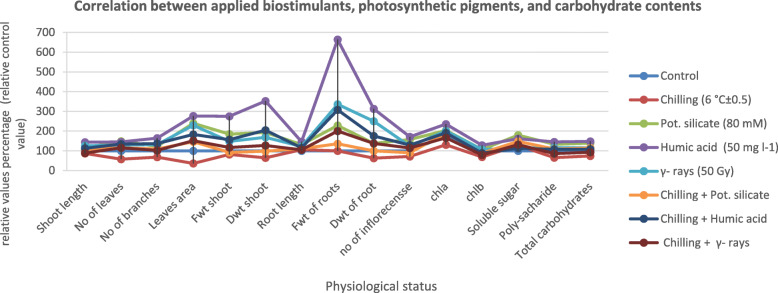
Correlation analysis linking the interaction between the application of biostimulants applied on the chilling-stressed on coriander (*Coriandrum sativum* L*.*) seeds pre- soaked in 80 mM pot. Silicate, 50 mg l^− 1^ humic acid or soaked in water after exposure to γ-rays (50 Gy) and the improvement in the photosynthetic pigments (μg/g D. wt. in coriander leaves) and carbohydrate contents (g/100 g D. wt.) at the vegetative and flowering stages. *. The data in this figure represent the relative values percentage in compare to the control for the results in Tables 2 and 5 (the value/control*100)

In the present investigation, soluble sugars were increased in leaves of the chilling-stressed coriander plant, while polysaccharides and total carbohydrate contents (Table 6) were decreased as compared to the control plant agreeing with the previous investigation of Azymi et al. [12]. The accumulation of total soluble sugars was reported as a fundamental component in chilling tolerance in many plant species in response to chilling stress. Soluble sugars might act as compatible solutes under chilling stress [12]. It was suggested that soluble sugars play crucial roles in osmotic adjustment, protection of specific macromolecules, and stabilization of membrane structures [13]. Soluble sugars are thought to interact with phospholipids polar head groups in membranes and to prevent membrane fusion [13]. In addition, sucrose and other sugars play a central role as signaling molecules that regulate the physiology, metabolism and development of plants [8]. The reduction in polysaccharides and total carbohydrates of leaves of chilling coriander plants were correlated with arrested growth rate and decrease in leaf photosynthetic pigments (Table S1; Fig. 4). Specifically, upon HA application, an ameliorative impact in growth, metabolism, and expression of Toc and RuBisCO complexes was triggered (Fig. 4). It might be concluded that cold stress might inhibit the photosynthetic activity and/or increase partial utilization of carbohydrates into the soluble sugars and metabolic products [8]. On the other hand, pre-soaking the seed of coriander plants in silicate, HA, or exposed to gamma radiation induced significant increases in soluble sugar, polysaccharides, and total carbohydrates (Table 6). These effects were much more pronounced by HA alone or in combination with chilling treatment. Similar result concerning the effect of low dose of gamma radiation (20 Gy) on increasing the carbohydrate contents were reported on onion and potatoe [75] as well as Lupine [46]. HA was found to cause the accumulation of soluble sugars concomitantly with the increase in polysaccharides content and total carbohydrates in wheat plants grown in newly reclaimed soil [14]. Also, silicon has promoted photosynthetic pigments and hence total carbohydrates were increased. It could be concluded that silicate, HA, and gamma radiation alone or in combination with chilling stress have played prominent role in alleviating the water dehydration status caused by chilling stress in coriander plant either via osmotic adjustment by increasing soluble sugars or by stabilizing the chloroplast membrane and enhancing the photosynthetic rate resulting in increased content of carbohydrate biosynthesis.

### Antioxidant compounds

Synthesis of compatible solutes de novo like osmoprotectants, sugars, amino acids, carotenoids, flavonoids, phenols, and polyphenols is regarded as adaptive plant mechanism against osmotic and oxidative stress [9]. The presented study has investigated significant decrease in ascorbic acid content by 46.81% below the control value caused by chilling stress (Table 7). Furthermore, all individual applied treatments or in combination with chilling stress have induced a significant increase in ascorbic acid content as compared with chilling-stressed plant. The most effective treatment alleviating the impact of chilling stress was HA (Table 7). These results are in harmony with those of Pokluda et al. [79] who reported significant increase in ascorbic acid, total phenolic concentration, and total antioxidant activity in chilled coriander downstream of biostimulants application. On the same context, reduction in carotenoids content was concomitant with significant increase in ABA level in stressed coriander leaves below the control. This might be speculated as an adaptive mechanism to stress. ABA biosynthesis from C_40_ carotenoids was assured to enable plants to cope with unfavorable condition [33, 104]. Notably, a marked increase in carotenoids content, downstream silicate and HA treatments, was most likely attributed to their antioxidant efficacy in trapping free radical and quenching singlet oxygen [81]. Latter results agreed with that of Habibi [29] who reported that silicon increases synthesis of protective pigment such as carotenoids and anthocyanin in chilling-stressed grapes. The investigated increase of phenols and flavonoids in the presented study, either upon chilling stress or after alleviation using silicate or HA treatments, was also reported by Rivero et al. [82] and then was attributed by Pokluda et al. [79]. The increase in proline content in chilling-stressed coriander leaves was higher than the control values. This might be due to induced synthesis and accumulation of compatible solutes such as proline or due to the inhibition of protein synthesis followed by increased level of free amino acids, especially proline [88]. In the present work, silicate, HA, and γ- rays and their interaction with chilling stress have induced a pronounced increase in proline content (Table 7). Latter findings were supported by Ahmad and Haddad [5] who worked on wheat and demonstrated the promoting effect of silicate on proline production under abiotic saline conditions. Moreover, HA and gamma radiation application on chilling-stressed coriander plant were shown to increase proline content. These results were similarly demonstrated on irradiated coriander [53], *Pisum sativum* L. [77], and wheat [14] plants.

### Antioxidant enzymes and lipid peroxidation

In this study, it was shown that chilling stress has caused a significant increase in CAT, PPO, and POD activities (Table 8). These results were in line with previous studies regarding CAT enzyme activity in maize seedlings [27] and other various plant species [50]. Inducing the activity of antioxidant enzymes by chilling stress is most likely regarded as a plant-derived defense mechanism to protect cell membranes, proteins, and metabolic machinery, which would preserve the subcellular structure from damage as a result of cell dehydration [85]. Alleviation of the chilling stress by γ- rays, has maintained and/or slightly increased the activity of PPO enzyme. A significant increase in the PPO enzyme activity was found using low doses of γ- radiation [43]. Furthermore, irradiation by γ- rays has increased the PPO and POX capacities in fresh fruits and vegetables [95]. Generally, activities of scavenging enzymes, such as POD, CAT, and SOD increased in various plant species in response to ionizing radiation [48, 101], especially the potential activity of POD to remove toxic H_2_O_2_. In the same context, silicon alleviates abiotic stress by enhancing the production of antioxidant enzymes involved in detoxifying free radicals [105]. It also increases their activities which in turn protect plants against ROS generation and lipid peroxidation [30]. The hindrance effect of induced activities of antioxidant enzymes to protect the cells from lipid peroxidation, caused by chilling stress, was evidenced by the reduction of MDA accumulation by all applied treatments. Latter investigation agreed with elsewhere previous studies [29, 64, 72, 85, 102] especially by using HA and silicate as stress alleviation elements.

### Significant expression of RuBisCO_LS_ and toc complex subunits in chilled alleviated coriander plants

Extracted TCPs were fractionated by SDS-PAGE technique. By achieving high-quality protein profiles, it was important to study and analyze the ameliorative effect of silicate, HA, and gamma irradiation on expressed TCPs generally and RuBisCO_LS_ expressed protein specifically. High variation in RuBisCO_LS_ expression level was revealed by chilling stress (Fig. 1a). Accumulation of RuBisCO_LS_ protein product, containing the active site, was demonstrated upon HA treatment [93]. Toc and RuBisCO enzyme complexes were detected at the same molecular weight demonstrated by Ladig et al. [51]. The complex activity was judged by the assembled RuBisCO complex in the cell. The biosynthesis/degradation rate of the RuBisCO two subunits controlled by gene expression is significantly affected by unfavorable abiotic conditions [49]. However, continuous significant accumulation of RuBisCO_LS_ may have a negative impact on the efficiency and the assembly of RuBisCO complex. Induced changes in the protein profiles of chilling stressed and alleviated samples by HA occurred within a narrow range (45–80 kDa) of polypeptides and were recorded in this study (Fig. S1a, b). On the same context, 25 protein spots were differentially and up-regulated in response to low temperature (4 °C) during imbibition in a known chilling-resistant soybean cultivar Z22 [20]. It had been found that optimum temperature for photosynthesis is 20 °C in barley [94]. Temperature stress has a deleterious effect on the photosynthesis apparatus [83]. In this context, the protein expression of the chloroplast coupling factor (CF1) was negatively affected by the chilling stress [45]. By grouping control and HA alleviated chilling-stressed plants together (Fig. 2), the cluster analysis has reflected the ability of HA treatment to alleviate the deleterious effect of chilling stress on the coriander plant proteostasis, especially RuBisCO_LS_. It might be concluded that chilling stress affects the photosynthesis process by disruption of RuBisCO complex assembly inside the chloroplast via down regulating the production of Toc machinery subunits (Toc34 and Toc75) and HSP70 chaperone. Latter impact would limit and restrict RubisCO import and assembly into chloroplast [41].

Taken together, it might be concluded that applied growth stimulators in this study, especially HA followed by silicate, have enhanced the antioxidative defense system for limiting the oxidative damage for coriander plants under chilling stress by scavenging excessive ROS through inducing non-enzymatic antioxidant compounds (ascorbic acid, carotenoids, total phenolic, flavonoids, and proline) as well as antioxidant defense enzymes (CAT, POD, and PPO). Besides that, molecular diagnosis of the catalytic effect of biomarkers to reduce the chilling stress at the level of TCPs was assigned and evidenced the restoration and maintenance of RuBisCO_LS_. Consequently, achieved improvements of growth parameters and yield components have reflected previous demonstrations. Hereby, presented results may reflect new insights and broaden our understanding about tolerance mechanism(s) against chilling stress in order to produce winter resistant crops of highly important economic importance like the coriander plant. This study has investigated the potential positioning of physiological, biochemical, and molecular analyses to evaluate and judge the effect of temperature stress fluctuations on the coriander crop in Egypt.

## Conclusions

Acclimation to chilling stress was reinforced in the coriander plant by priming of coriander seeds in potassium silicate (80 mM), humic acid (50 mg. l−^1^) or priming in water after being exposed to gamma rays (50 Gy) and their combination with chilling stress. Alleviated chilling stress was characterized in coriander by improved plant growth and decreased ABA level. Photosynthetic pigments and carbohydrates content (c.a. soluble sugars) were positively promoted concomitantly with polysaccharides and total carbohydrates after alleviation of chilling stress using applied growth stimulators. Moreover, investigated antioxidants compounds and enzymes have undergone either induction or significant increase upon pre- and alleviation treatments. Besides that, induction the accumulation of large subunit of RuBisCO enzyme was also reported as a sign for restoration and maintenance of cellular protein homeostasis. Therefore, it could be suggested that the effectiveness of biostimulators used in this study (especially HA) and their potential stimulatory effect has induced stress tolerance in cultivated coriander under low temperature. The biostimulators applied in the presented study most likely triggered a pronounced step to enhance acclimation and tolerance of coriander plant to chilling stress by safe methods, thus improving and stimulating bioactive hormones, pigments, and healthy components.

## Methods

### Plant material and applied treatments

Coriander (*Coriandrum sativum* L.) seeds used in this study was assessed by Agricultural Research Center (ARC), Ministry of Agriculture, Giza, Egypt, purchased from seeds suppliers’ in Egyptian local market by Abd Elhady Gayar Company, Cairo, Egypt, and named by “Baladi variety”. The HA used in this study is produced and purchased from Misr International Company for Agricultural and Industrial Development, Cairo, Egypt. This product is registered and accredited under the name of “HUMO” with No. 7050, Egyptian Ministry of Agriculture, Cairo, Egypt. The prementioned HA product was approved from Agriculture Research Center (ARC), Giza, Egypt. Potassium silicate (99% degree of purity) was purchased from Sigma-Aldrich Company (Cat. No. 792640). Pilot experiments and basic aspects of the optimization process were carried out with a wide range of potassium silicate or humic acid concentrations (like sub-optimum, optimum, and supra-optimum concentrations). To detect the optimum concentration of HA, various ascending concentration were applied; 5, 10, 25, 50, 75, and 100 mg.l^− 1^. The best concentration was 50 mg.l^− 1^. In case of potassium silicate, a series of concentrations; 10, 20, 40, 80, and 160 mM were used. It was found that 80 mM is the optimum concentration. The judgement of the results’ quality in the stage of executing the pilot experiments was based on the highest records of growth parameters and yield components. Then, these experimental results were obtained and provided us with a solid basis to which optimum concentration should be selected. The used water source, named as “tap water” in this study, met the standard requirements of WHO (World Health Organization, Geneva 2008). The needed details of the water analysis were accompanied as a supplementary data set. Seed priming was performed by tap water using solutions of potassium silicate (80 mM) or humic acid (50 mg.l^− 1^) prior to seeds chilling (6.0 ± 0.5 °C) or non-chilling (20.0 ± 2.0 °C) conditions for 16 h in water. Similarly, coriander dry seeds were irradiated using gamma rays (50 Gy) prior to rinsing in non-chilled or chilled water for 16 h. The irradiation experiment for chilled and non-chilled seeds was carried out in National Center for Radiation Research and Technology (NCRRT), Atomic Energy Authority, Cairo, Egypt using Cesium-137 with a dose rate 0.758 rad/sec. The experiment was carried out during two successive seasons; a short description of experimental protocol is presented and listed in Table 1.

Soaked seeds were washed thoroughly with distilled water, then sown in field plastic pots (L .W .D = 50 × 50 × 80 cm) containing 15 Kg clay: sandy soil (2:1 w/w), ten seeds/pot, and 10 pots for each treatment. The number of pots were counted putting into consideration that sample collection was planned to be performed at different growth and developmental stages. Pots were irrigated by tap water to keep 80% water holding capacity. Plants of the vegetative stage were harvested at day 75 from the sowing date, while, the plants of the flowering stage were harvested after 105 days. Yield components were harvested after 135 day from the sowing date. Throughout this study, three biological and/or three technical replicates were used to measure either growth/yield parameters or to perform chemical and molecular analyses. Representative samples of ten plants (one pot; counted as one biological replicate) were taken from each treatment at (vegetative stage and flowering stage) to measure the growth parameters; plant height, root length, number of branches /plant, number of leaves/plant, area of leaves/plant, and fresh and dry weights of shoot and root/ plant. Yield components parameters (number and weight of seeds/ plant as well as seed index) were recorded for each treatment. Chemical analyses were carried out in coriander leaves at flowering stage. The experiments were repeated at the next season and the mean values of growth parameters and yield components were recorded. The experiment design was completely randomized.

### Extraction, separation and estimation of growth regulating substances

The method of extraction was identical to that adopted by Shindy and Smith [87] and described by Hassanein et al. [34]. Determination and identification of acidic hormones (IAA, GA3, and ABA) were performed as described by Kelen et al. [44]. The plant tissues (five grams of each sample out of the three independent used technical replicates) were collected and ground in 80% methanol. The macerated tissues were transferred to a flask with fresh methanol and the volume was adjusted to 20 ml of methanol for each g fresh weight of sample. The tissues were extracted for 24 h at 0 °C and then was vacuum filtrated through Whatman filter paper (No. 42). The residues were returned to flask with fresh volume of methanol and stirred for 30 min with magnetic stirrer and then filtrated again. The procedure was repeated once more and combined extract ions were evaporated to the aqueous phase in a rotary flash evaporator. The aqueous phase (10–30 ml) was adjusted to pH 8.6 with 1% (w/v) NaOH and partitioned three times with equal volumes of ethyl acetate. The combined ethyl acetate fraction was evaporated to dryness and held for further purification. The aqueous phase was adjusted to pH 2.8 with 1% HCl (v/v) and re- partitioned three times with equal volume of ethyl acetate. The remaining aqueous phase was discarded, and the combined acidic ethyl acetate phase was reduced to 5 ml (fraction I) to be used for gas chromatography (GC) determination of acidic hormones such as IAA, ABA and GA_3_. To estimate the amounts of acidic hormones (fraction I) by HPLC isocratic UV analyzer, reverse phase C18 column (RO-C18 μBondapak, WATERS corporation, MA, USA) was detected. The column used included octadecylsilane (ODS) ultrasphere particle (5 μm), the mobile phases used were acetonitrile-water (26:74 v/v), PH 4.00; Flow rare: 0.8 ml/min, detection by UV at 208 nm, the standard solution of the individual acid was prepared in mobile phase and chromatographed. The retention times of peaks of authentic samples were used in identification and characterization of peaks of samples under investigation. Peak identification was performed by comparing the relative retention time of each peak with those of IAA, GA_3_, and ABA standard. Peak area was measured by triangulation and relative properties of the individual component were therefore obtained at various retention times of samples.

### Estimation of photosynthetic pigments

The photosynthetic pigments; chlorophyll a (chl *a*), chlorophyll b (chl *b)*, and carotenoids were determined by Metzner et al. [69]. Briefly, fresh weight of leaves (0.5 g) was homogenized in 85% aqueous acetone for 5 min. The homogenate was centrifuged, and the supernatant was made up to volume with 85% aqueous acetone. The extinction was measured against a blank of pure 85% aqueous acetone at 3 wave lengths of 452.5, 644, and 663 nm using Spectropolarimeter DC Tiny 25III Model TUDC12B4. The photosynthetic pigments were determined in μg/ml using the following equations: *Chlorophyll a = 10.3 E663–0.918 E644, Chlorophyll b = 19.7 E644–3.87 E663, and Carotenoids = 4.2 E425.5 - (0.026 chlorophyll a + 0.426 chlorophyll b).* Finally, the pigment contents were expressed in μg.g^− 1^ of leaves dry weight.

### Estimation of carbohydrates

For soluble sugars and polysaccharides determination, plant material (one gram of fresh tissue) was oven-dried at 80 °C to constant weight and then ground to a fine powder using local domestic blender. For extraction and estimation of soluble sugars, 25 mg of dried tissues was homogenized using 80% ethanol, and then kept in boiling water bath with continuous shaking for 15 min. After cooling, the extract was filtrated, and the filtrate was oven dried at 60 °C then dissolved in 2 ml of water to be ready for determination of soluble sugars [40]. The anthrone sulphuric acid method carried out by Whistler et al. [100] was used for determination of soluble sugars. Polysaccharides were extracted and estimated using the dry residue left after extraction of soluble carbohydrate. A known weight of dried material (100 mg) was added to 10 ml 1.5 N sulfuric acid in sugar tubes with air reflux at 100 °C in a water bath for 6 h. Then, the hydrolysate was neutralized by 2.5 N NaOH using phenol red as indicator. The latter neutralized solution was used for polysaccharide determination by method of anthrone sulphuric acid reagent [37, 100]. A calibration curve using pure glucose was made, from which the data were calculated as mg/g dry weight. Finally, total carbohydrates content was expressed as the summation of soluble sugars plus polysaccharides amounts in each sample.

### Extraction and estimation of antioxidants compounds

In this study, the antioxidants defense compounds (ascorbic acid, total flavonoids, phenolic compounds, and proline content) were determined. Ascorbic acid was determined in mg/100 g fresh leaves by 2,6 dichlorophenol indophenol for titration according to Zvaigzne et al. [106]**.** Briefly, ten grams of leaves were accurately weighed and ground using mortar and pestle with an additional of 20 ml of 3% metaphosphoric acid- acetic acid solution. The mixture was further ground and strained through muslin and the extract was made up to 100 ml with the metaphosphoric-acetic acid mixture. Five ml of the metaphosphoric acid-acetic acid solution was pipetted into three of the 50 ml Erlenmeyer flask followed by 2 ml of the samples extract. The samples were titrated separately with the indophenol dye solution until a light rose pink persisted for 5 s. The amount of dye used in the titration were determined and used in the calculation of vitamin C content. Total flavonoids contents were determined by the aluminum chloride colorimetric assay according to Marinova et al. [66]. Each ethanolic extract (1.0 ml) or standard solution of quercetrin was added to 10 ml volumetric flask containing 4.0 ml distilled water. To the flask 0.3 ml of 5% NaNO_2_ was added. After 5 min, 0.3 ml of 10% AlCl_3_ was added and after 6 min, 2.0 ml 1 M NaOH was added and the total volume was made up to 10 ml with distilled H_2_O. The solution was mixed, and the absorbance was measured against the blank at 510 nm. Finally, total flavonoids were expressed as mg quercetin equivalent per 100 g of dry weight. Moreover, phenolic compounds were estimated according to Malik and Singh [65] in which phenols could react with phosphormolbdic acid in Folin-Ciocalteau reagent in alkaline medium and produce blue colored complex (molybdenum blue). The absorbance was measured using Milton Roy Spectronic 601 Spectrophotometer at 650 nm. The concentration of phenolic compounds per 100 g leaves (fresh weight) was calculated from gallic acid standard curve. The values were then calculated as (mg 100 g^− 1^) dry weight. Free proline was determined according to the method of Bates et al. [15]. This method was based on the reaction between proline and acid ninhydrin reagent. Acid ninhydrin reagent was prepared by warming 1.25 g ninhydrin in 30 ml glacial acetic acid and 20 ml 6 M phosphoric acid with agitation until dissolved; kept cool and stored at 4 °C. The reagent remains stable for 24 h. Approximately 0.1 g of macerated dried tissue was homogenized in 10 ml of 3% aqueous sulfosalicylic acid, and then filtered through filter paper Whatman No. 2. Two ml of the filtrate were mixed with 2 ml glacial acetic acid and 2 ml of the acid ninhydrin reagent in a test tube and heated for 1 h at 100 °C .The reaction mixture was extracted with 4 ml toluene, mixed vigorously in a test tube for 15–20 s. The chromophore containing toluene was aspired from the aqueous phase and warmed to room temperature. The absorbance was read at 520 nm using toluene as a blank. The proline concentration was determined using stander curve and calculated on a dry matter basis.

### Extraction and measurements of antioxidants enzymes

The antioxidants enzymes (catalase (CAT), peroxidase (POD), and polyphenol oxidase (PPO)) were extracted from frozen ground leaves (0.5 g) using cold mortar and pestle and homogenized with cold sodium phosphate buffer (100 mM, pH = 7) containing 1% (w/v) polyvinylpyrrolidone (PVP) and 0.1 mM EDTA. The extraction ratio was 4 ml extraction buffer for each one gram of plant tissues. The homogenate was centrifuged at 10,000 g at 4 °C for 15 min. The supernatant was used for to measure CAT, POD, and PPO activities. Also, protein concentration was quantified in the crude extract by Lowry et al. [58] using bovine serum albumin as a standard. The activity of CAT (EC 1.11.1.6) was determined according to Aebi [3]. Enzyme extract (100 μl) was added to 2.9 ml of a reaction mixture containing 20 mM H_2_O_2_ and 50 mM sodium phosphate buffer (pH 7.0). The activity of CAT was measured by monitoring the reduction in the absorbance at 240 nm as a result of H_2_O_2_ consumption. The amount of consumed H_2_O_2_ was calculated by using a molar extinction coefficient of 0.04 cm^2^ μmol^− 1^. One unit of enzyme activity was defined as the decomposition of 1 μmol of H_2_O_2_ /min. Catalase activity was expressed as unit min^− 1^ mg^− 1^ protein. Also, POD (EC1.11.1.7) activity was quantified by the method described by Hammerschmidt et al. [31]. The assay mixture (100 ml) contained 10 ml of 1% (v/v) guaiacol, 10 ml of 0.3% H_2_O_2_ and 80 ml of 50 mM phosphate buffer (pH = 6.6). Volume of 100 μl of crude enzyme was added to 2.9 ml of the assay mixture to start the reaction. The absorbance was recorded every 30 s for 3 min at 470 nm using spectrophotometer (UV-Vis spectrophotometer UV 9100 B, LabTech). The rate of change in absorbance per minute was calculated and one unit of enzyme was expressed as ΔOD = 0.01. The POD activity was expressed as (unit min^− 1^ mg^− 1^ protein). Moreover, PPO (EC 1.14.18.1) activity was measured according to Oktay et al. [76]. The reaction mixture contained 600 μl catechol (0.1 M) and 100 μl enzyme extract was completed to 3.0 ml with 0.1 M phosphate buffer pH 7. The absorbance was recorded at 420 nm by spectrophotometer (UV-visible-160A, Shimadzu). One unit of PPO activity was defined as the amount of enzyme that causes an increase in absorbance of 0.001 min^− 1^ ml^− 1^. The enzyme activity was expressed as (unit min^− 1^ mg^− 1^ protein).

### Estimation of lipid peroxidation

The lipid peroxidation in fresh coriander leaves was determined by measuring the amount of Malondialdehyde (MDA) produced by thiobarbituric acid (TBA) reaction as described by Heath and Packer [36]. The leaves (0.5 g) were homogenized in 5 ml of 0.1% (m/v) TCA. The homogenate was centrifuged (Hettich Zentrifugen Universal 16 R Centrifuge, Hettich Rotor 1612 12X3g, Germany) at 10,000 g for 20 min. To an aliquot (1 ml) of the supernatant, 4 ml of 0.5% TBA in 20% TCA was added. The mixture was heated at 95 °C water bath for 30 min and then quickly cooled in an ice bath. After centrifugation at 10, 000 g for 15 min, the absorbance of the supernatant was recorded at 532 and 600 nm. The value for non-specific absorption at 600 nm was subtracted. The concentration of MDA was calculated by dividing the difference of (A532-A600) by its molar extinction coefficient (155 mM^− 1^ cm^− 1^) and the result was expressed as (nmol g^− 1^) fresh weight.

### Extraction of total cellular proteins (TCPs) and chloroplast protein complexes from coriander

TCPs and chloroplast protein complexes were extracted from coriander leaves at the vegetative stage (75 days old) according to Mehta et al. [68] with minor modifications. Briefly, chilling stress-primed coriander leaves were ground in liquid nitrogen to a fine powder using the mortar and pestle. To assure complete homogenous cellular disruption, aliquots (250 mg) were subjected to high throughput TissueLyser II equipment (Qiagen, Cay. No. 85300) for three times/30 s each. Immediately, extraction buffer (100 μl) of 100 mM Tris-HCl PH 8, 50 mM EDTA, 40% Glycerol, 4% β-mercaptoethanol, 2% w/v SDS, 0.1 mM phenylmethylsulfonyl fluroide (PMSF), 1x protease inhibitors cocktail (Roche, Penzberg, Germany), and 0.001% bromophenol blue dye was added to the ground leaves and mixed until a completely homogeneous lysate is obtained using the mortar and pestle. The tissue lysate was vortexed for three minutes, incubated at 95 °C for 5 min (Eppendorf™ Thermomixer™), and finally centrifuged (Hettich MICRO 22 centrifuge, Germany) at high speed for 30 min at 20.000 xg. Subsequently, the supernatant was removed and saved as 25 μl aliquots at − 80 °C freezer for further analysis by SDS-PAGE technique. Detection of protein concentration was performed using protein assay dye reagent (BioRad, cat No. #5000006). Bradford programmed method (Eppendorf Biophotometer, ver. 1.35, Model #6131) and calibration memory for protein methods were used to quantify the protein concentration according to Bradford [18].

### SDS−/ HDN-PAGE and immunoblotting techniques

Previously extracted TCPs were subjected to either preparatory and/or analytical one-dimensional 10% SDS-PAGE or gradient 4–12% HDN-PAGE procedures as previously described [51, 52, 68]. Color-coded pre-stained protein marker (High-Range SDS-PAGE Standards, GeneON, Ludwigshafen, Germany) was loaded and electrophoretic fractionation was carried out using Bio-Rad Mini-Protean II Cell Gel System at 70 V in 1 X premade tank buffer (BioRad, #1610734). Protein extraction procedure for each physiological status (control, chilling-stressed, etc.) was performed from the leaves of 3 biological replicates and 3 technical replicates. Each technical replicate represented one biological replicate. Each biological replicate comprises the collection of leaves of 10 plants. The protein extraction was carried out from each technical replicate independently. Finally extracted proteins from the 3 technical replicates were pooled together. Pooled sample was quantified and equally loaded into 10% SDS-PAGE consequently after measuring its concentration. Aliquots of pooled sample were kept as − 80 °C after the short snap for 30 s in Liquid Nitrogen. After completion of protein migration, the gel was stained, de-stained, and finally placed between two sheets of cellophane membrane for immunoblotting technique and/or for documentation purposes as demonstrated by Ladig et al. [51]. Gel images were captured using Bio-Rad gel documentation system (Gel Doc™ EZ system and enabled Image Lab™ software). Protein concentration was revealed using normalized Bovine serum albumin (BSA) standard curve. The cluster analysis was constructed by the following; Band Scoring {(0) for absence and (1) for presence}, based on SDS-PAGE fractionated protein profile, was performed. The binary matrix was generated using the data as revealed by SDS-PAGE. The binary matrix was executed to calculate the genetic similarity matrix coefficient. A distance tree was constructed using the unweighted pair group method with arithmetic mean (UPGMA) using PAST, ver. 4.02 as previously described by Hammer et al. [38]. Blotting onto a PVDF membrane (0.1 μ, Schleicher & Schull, Germany) in Towbin-buffer (192 mM Glycin, 25 mM Tris/HCl, pH 8.3, 0,1% (w/v) SDS, and 15% (v/v) Methanol) was carried out using Bio-Rad Trans-Blot® Semi-Dry electrophoretic cell (Cat. number 170–3940) according to manufacturer’s instructions. Phosphate buffered saline (PBS), supplemented with Tween-20, were used for membranes washing steps intervening the primary and secondary antibodies incubation times. Monoclonal primary antibodies against *At*Toc75 and *At*Toc34 (*A. thaliana* chloroplast outer membrane proteins with 75 and 34 kDa, respectively), eukaryotic HSP70 (eHSP70; intermembrane space chaperone for RuBisCO translocation into chloroplast inner membrane), plant Actin (as a control housekeeping gene), and HRP-conjugated secondary anti-rabbit IgG were used. All primary and secondary antibodies were used at dilutions 1: 10,000 and 1: 25,000, respectively and purchased from Agrisera (Vännäs, SWEDEN). Immunoblotting (western blotting, WB), detection of immobilized specific antigens conjugated to Horseradish Peroxidase (HRP), and visualization of HRP was executed by chemiluminescent (ECL) detection kit (Pierce™ ECL Western Blotting Substrate, ThermoFisher SCIENTIFIC, Cat. No. 32106**)** according to the manufacturer’s recommendations. Moreover, by using of ImageJ software (IJ 1.46r) for image processing and analysis of the electrophoretic running of ascending concentration series of Bovine serum albumin (BSA), used as protein size standard, quantification of RuBisCO large subunit (RuBisCO_LS_) and Toc (Translocon at outer membrane of chloroplast) complex band were performed.

### Statistical analysis

The experimental procedure for each physiological status (control, stress, etc.) was performed from the leaves of 3 biological replicates and 3 technical replicates. Each technical replicate represented the mean of one biological replicate members. Each biological replicate comprises the collection of plant tissue or leaves of 10 plants (one pot). The mean of the independent technical replicates was calculated, and the mean values were used to calculate ±SE. The data were statistically analyzed for variance and the values of least significant differences (LSD) at 5% level were calculated to compare the means of different treatments according to Snedecor and Cochran [92]. Different letters indicate significant variation according to Duncan^’^s multiple rang test to discriminate significance (defined as *P* < 0.05).

## Supplementary Information

**Additional file 1: Suppl. Fig. 1.** Impact of alleviation treatments on growth parameters of chilling-stressed coriander plants at the flowering stage. **Suppl. Fig. 2.** Impact of alleviation treatments on TCPs profiles of chilling-stressed (6 °C ± 0.5) coriander plants at the vegetative stage (75 days old). Protein extraction procedure for each physiological status (control, stress, etc.) was performed from the leaves of 3 biological and 3 technical replicates. Each technical replicate represented one biological replicate. Each biological replicate is composed of the collection of leaves of 10 plants. The latter collected leaves represented one technical replicate. The protein extraction was carried out from each technical replicate independently. Finally extracted proteins from the 3 technical replicates were pooled together. Pooled sample were quantified and equally loaded into 10% SDS-PAGE consequently after measuring its concentration. Aliquots of pooled sample were kept as − 80 °C after the short snap for 30 s in Liquid Nitrogen. Coriander control (Lane 1) seeds and chilling-stressed (Lane 2) ones were subjected to pre- soaking in 80 mM Pot. silicate (Lane 3), 50 mg l^− 1^ HA (Lane 4) or soaked in water after exposed to 50 Gy gamma irradiation (Lane 5). TCPs were then extracted, fractionated on 10% SDS-PAGE for season 1 (**Panel a**) and season 2 (**Panel b**), and finally stained with CoBB stain. The numbers shown on the left-handed side of the figures indicate molecular weight standards in kDa (High-Range SDS-PAGE Standards, GeneON, Ludwigshafen, Germany). Red arrowheads refer to induced upregulated polypeptides detected in “chilling+HA”, but not in “Chilling” and/or other chilling plus alleviation elements. The asterisk refers to approximate molecular weight of RuBisCO_LS_. ImageJ software (IJ 1.46r) was used for image processing and analysis of the electrophoretic running of ascending concentration series of BSA (as protein size standard) to quantify RuBisCO_LS_ concentration in (μg) downward of every panel. Full-length gels are presented in Supplementary Fig. (2a and b). Black arrowhead refers to the protein band at approximate molecular weight of 180 kDa used for normalization of quantified RuBisCO _LS_ protein expression. Suppl. Fig. 3 Full-length original and unprocessed Immunoblot analysis of the expression of chloroplast marker proteins. TCPs were extracted and fractionated by SDS-PAGE and immunodecorated against α-Toc34, α-Toc75, eHSP70, and actin primary antibody in a dilution of 1:10,000 as demonstrated in [51]. Cropping of the shown blots was performed properly for sake of clarity and focusing the information. Full-length blots are accompanied the manuscript as **Supplementary Fig. 3.** Protein extraction procedure for each physiological status (control, chilling stressed, etc.) was performed from the leaves of 3 biological replicates and 3 technical replicates. Each technical replicate represented one biological replicate. Each biological replicate comprises the collection of leaves of 10 plants. The protein extraction was carried out from each technical replicate independently. Finally extracted proteins from the 3 technical replicates were pooled together. Pooled sample were quantified, equally loaded into 10% SDS-PAGE, and blotted onto PVDF membrane as shown in methods section. Consequently, aliquots of pooled sample were kept as − 80 °C after the short snap for 30 s in Liquid Nitrogen. Suppl. Fig. 4 Fractionation of chloroplast protein complexes by HDN-PAGE technique profiles of chilling-stressed (6 °C ± 0.5) coriander plants at the vegetative stage. **Panel** (**a**) Coriander control (Lane 2, 7) seeds and chilling-stressed (Lane 3) ones were subjected to pre- soaking in 80 mM Pot. silicate (Lane 5), 50 mg l^− 1^ HA (Lane 5) or soaked in water after exposed to 50 Gy gamma irradiation (Lane 6). Extracted protein complexes (especially RuBisCO enzyme complex) were then fractionated on 4–12% gradient native HDN-PAGES, and finally stained with CoBB stain. Native molecular weight standards (HMW Native Marker kit, GE Healthcare) (lane 1) was loaded and denoted by numbers left-handed of the figure indicating molecular weight standards in kDa. ImageJ software (IJ 1.46r) was used for image processing and analysis of the electrophoretic running of ascending concentration series of BSA (as protein size standard) to quantify RuBisCO_LS_ concentration and Toc complex bands in (μg) were depicted downward of the figure. Cropping of the HDN-PAGE shown was performed for sake of clarity and focusing the information. Full-length original HDN-PAGE gel is presented in Supplementary Fig. 4 (Repeat 1, 2). Black arrowhead refers to the protein band at approximate molecular weight of 300 kDa used for normalization of quantified Toc and RuBisCO protein complexes. Original full length HDN-PAGEs; Repeat 1 (**Panel a)** and Repeat 2 (**Panel b)** were shown without cropping for sake of clarity. Repeat 1 and 2 were executed to manifest the consistency and reproducibility of the given protein complexes. Panel (b) specifically showed also the loading of different concentrations of solubilized protein complexes of control sample for optimization and high-resolution purposes.

**Additional file 2.**

**Additional file 3: Supplementary Table 1.** Correlation matrix linking the interaction between the application of biostimulants applied on the chilling-stressed on coriander (*Coriandrum sativum* L*.*) seeds pre- soaked in 80 mM pot. Silicate, 50 mg l^− 1^ humic acid or soaked in water after exposure to γ-rays (50 Gy) and the improvement in both the photosynthetic pigments (μg/g D. wt. in coriander leaves) and carbohydrate contents (g/100 g D. wt.) at the vegetative and flowering stages. *. Correlation is significant at the 0.05 level (2-tailed). **. Correlation is significant at the 0.01 level (2-tailed).

## Data Availability

All datasets generated and/or analyzed during this study were completely included within the article and its supplementary information.

## Abbreviations

RuBisCO: Ribulose-1 5-bisphosphate carboxylase/oxygenase
RuBisCO_LS_: RuBisCO complex large protein subunit
IAA: Indole Acetic Acid
ABA: Abscisic Acid
HA: Humic Acid
Gy: Gray unit
HPLC: High performance thin layer chromatography
HDN-PAGE: Histidine Deoxycholate Native - Polyacrylamide Gel Electrophoresis
CAT: Catalase
POD: peroxidase
PPO: polyphenol oxidase
Toc: Translocon at the outer envelope membrane of chloroplast
