# Supplementary material for: Metabolic activities and molecular investigations of the ameliorative impact of some growth biostimulators on chilling-stressed coriander (Coriandrum sativum L.) plant
Source: BMC Plant Biol. 2021 Aug 7;21:361. doi: 10.1186/s12870-021-03021-6 (PMC8349021; doi:10.1186/s12870-021-03021-6)
Supplement: Supplementary file 1 — Additional file 1: Suppl. Fig. 1. Impact of alleviation treatments on growth parameters of chilling-stressed coriander plants at the flowering stage. Suppl. Fig. 2. Impact of alleviation treatments on TCPs profiles of chilling-stressed (6 °C ± 0.5) coriander plants at the vegetative stage (75 days old). Protein extraction procedure for each physiological status (control, stress, etc.) was performed from the leaves of 3 biological and 3 technical replicates. Each technical replicate represented one biological replicate. Each biological replicate is composed of the collection of leaves of 10 plants. The latter collected leaves represented one technical replicate. The protein extraction was carried out from each technical replicate independently. Finally extracted proteins from the 3 technical replicates were pooled together. Pooled sample were quantified and equally loaded into 10% SDS-PAGE consequently after measuring its concentration. Aliquots of pooled sample were kept as − 80 °C after the short snap for 30 s in Liquid Nitrogen. Coriander control (Lane 1) seeds and chilling-stressed (Lane 2) ones were subjected to pre- soaking in 80 mM Pot. silicate (Lane 3), 50 mg l− 1 HA (Lane 4) or soaked in water after exposed to 50 Gy gamma irradiation (Lane 5). TCPs were then extracted, fractionated on 10% SDS-PAGE for season 1 (Panel a) and season 2 (Panel b), and finally stained with CoBB stain. The numbers shown on the left-handed side of the figures indicate molecular weight standards in kDa (High-Range SDS-PAGE Standards, GeneON, Ludwigshafen, Germany). Red arrowheads refer to induced upregulated polypeptides detected in “chilling+HA”, but not in “Chilling” and/or other chilling plus alleviation elements. The asterisk refers to approximate molecular weight of RuBisCOLS. ImageJ software (IJ 1.46r) was used for image processing and analysis of the electrophoretic running of ascending concentration series of BSA (as protein size standard) to quantify RuBisCOLS concentratio [file 12870_2021_3021_MOESM1_ESM.pdf]

Supplementary Materials

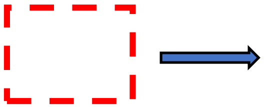 Refers to the position of performed cropping

Supplementary Figure 1

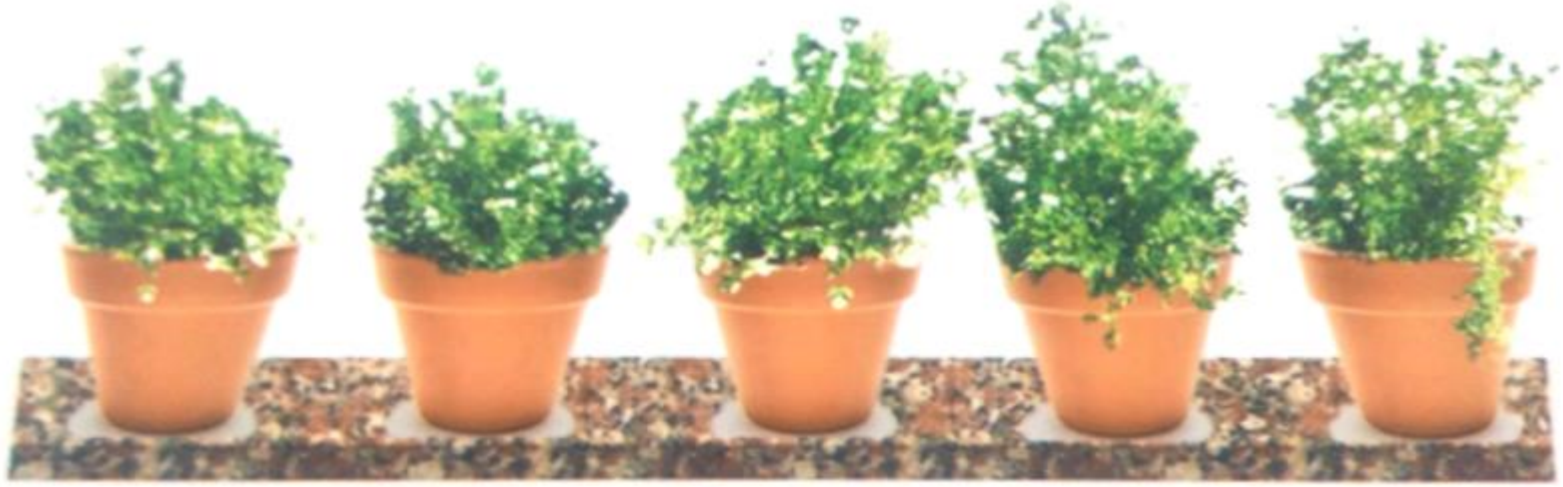

Control

Chilling (6°C±0.5)

Chilling + Pot. silicate

Chilling + Humic acid

Chilling + γ-irradiation

Supplementary Figure 2

Original and unprocessed version

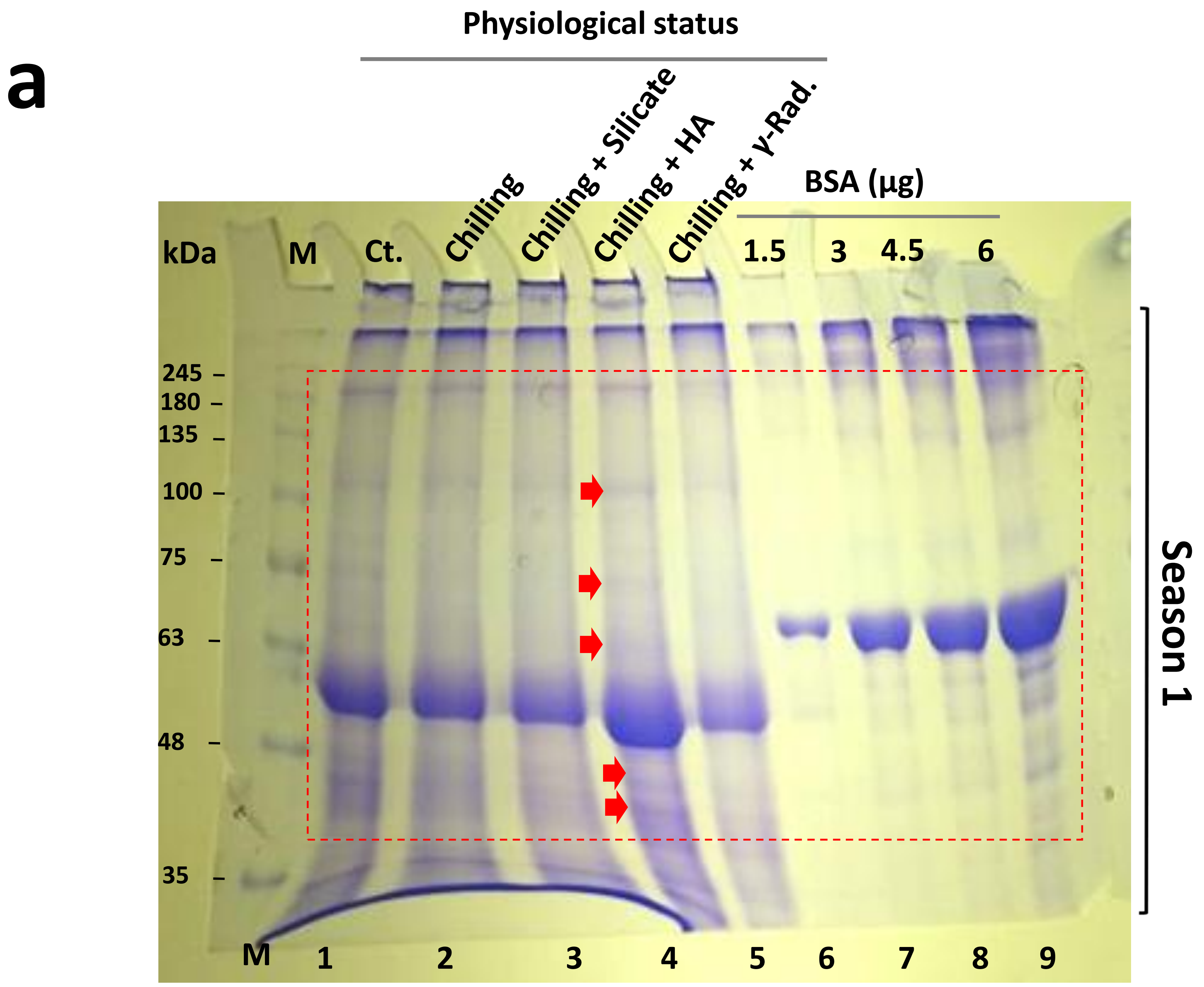
a
Physiological status
kDa
M
Ct.
Chilling
Chilling + Silicate
Chilling + HA
Chilling + γ-Rad.
BSA (µg)
1.5
3
4.5
6
245
180
135
100
75
63
48
35
M
1
2
3
4
5
6
7
8
9
Season 1

Processed version

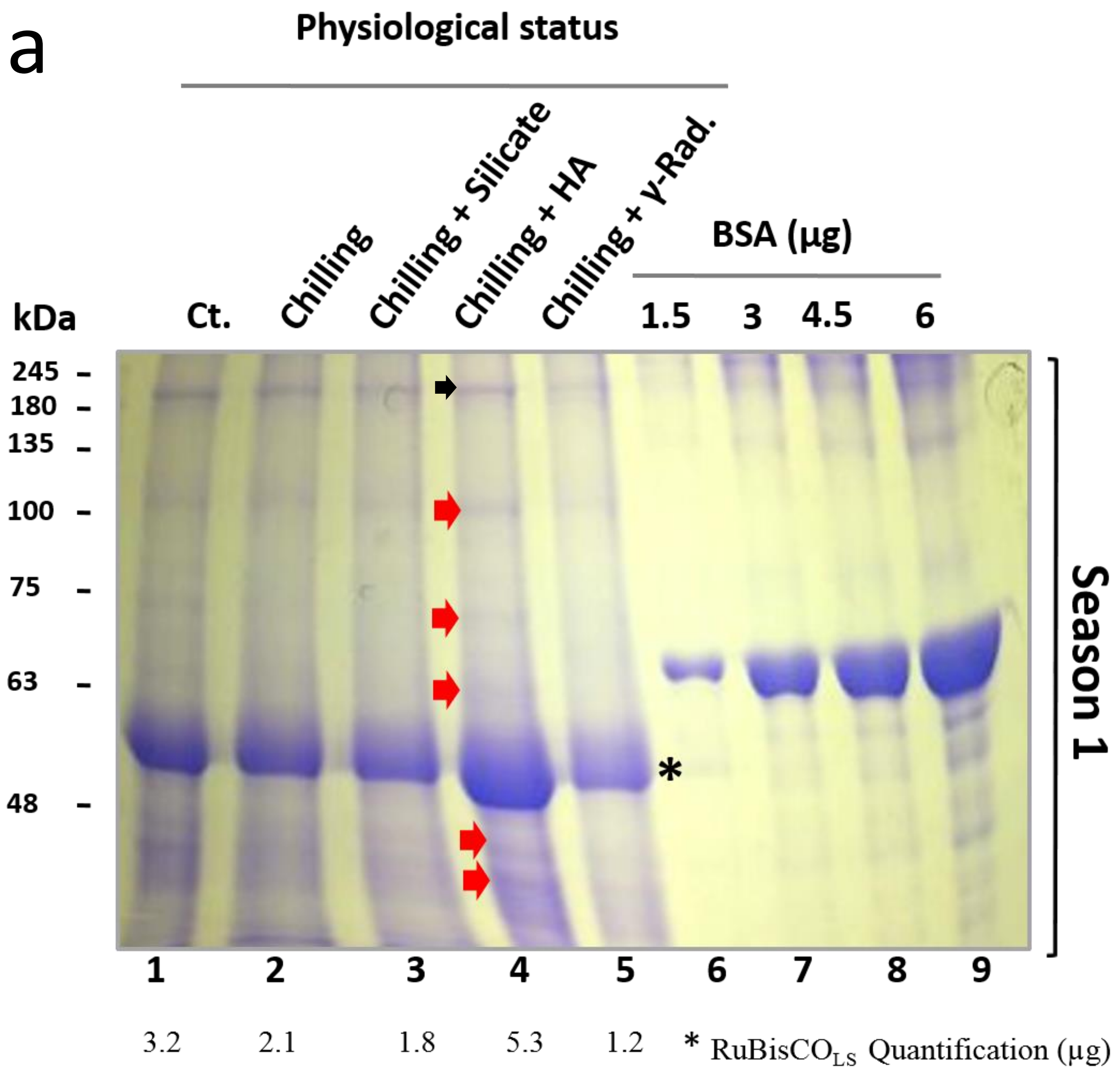
a
Physiological status
kDa
Ct.
Chilling
Chilling + Silicate
Chilling + HA
Chilling + γ-Rad.
BSA (µg)
1.5
3
4.5
6
245
180
135
100
75
63
48
Season 1
1
2
3
4
5
6
7
8
9
3.2
2.1
1.8
5.3
1.2
* RuBisCO$_{LS}$ Quantification (µg)

Original and unprocessed version

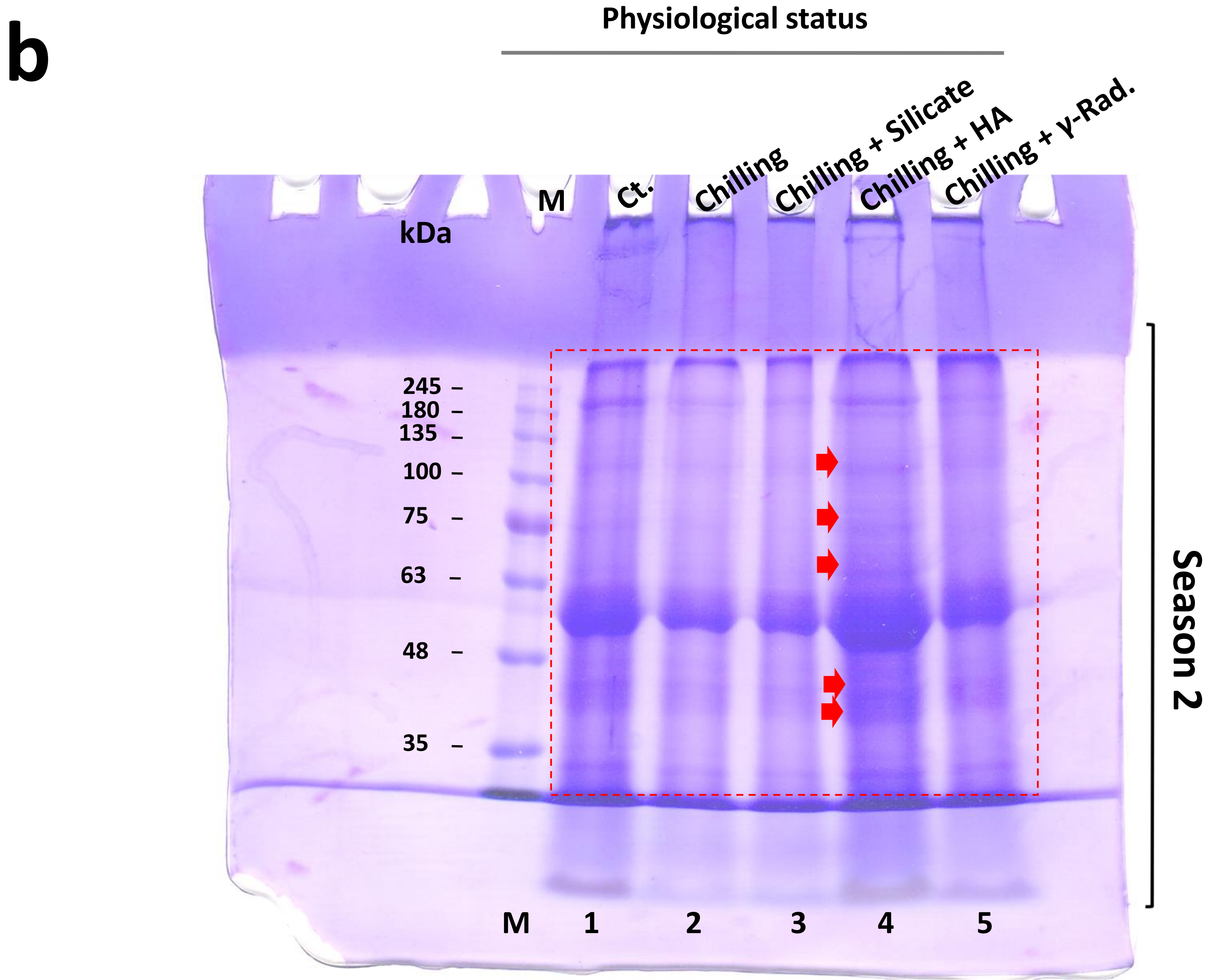

b
Physiological status
M
Ct.
Chilling
Chilling + Silicate
Chilling + HA
Chilling + γ-Rad.
kDa
245
180
135
100
75
63
48
35
M
1
2
3
4
5
Season 2

Processed version

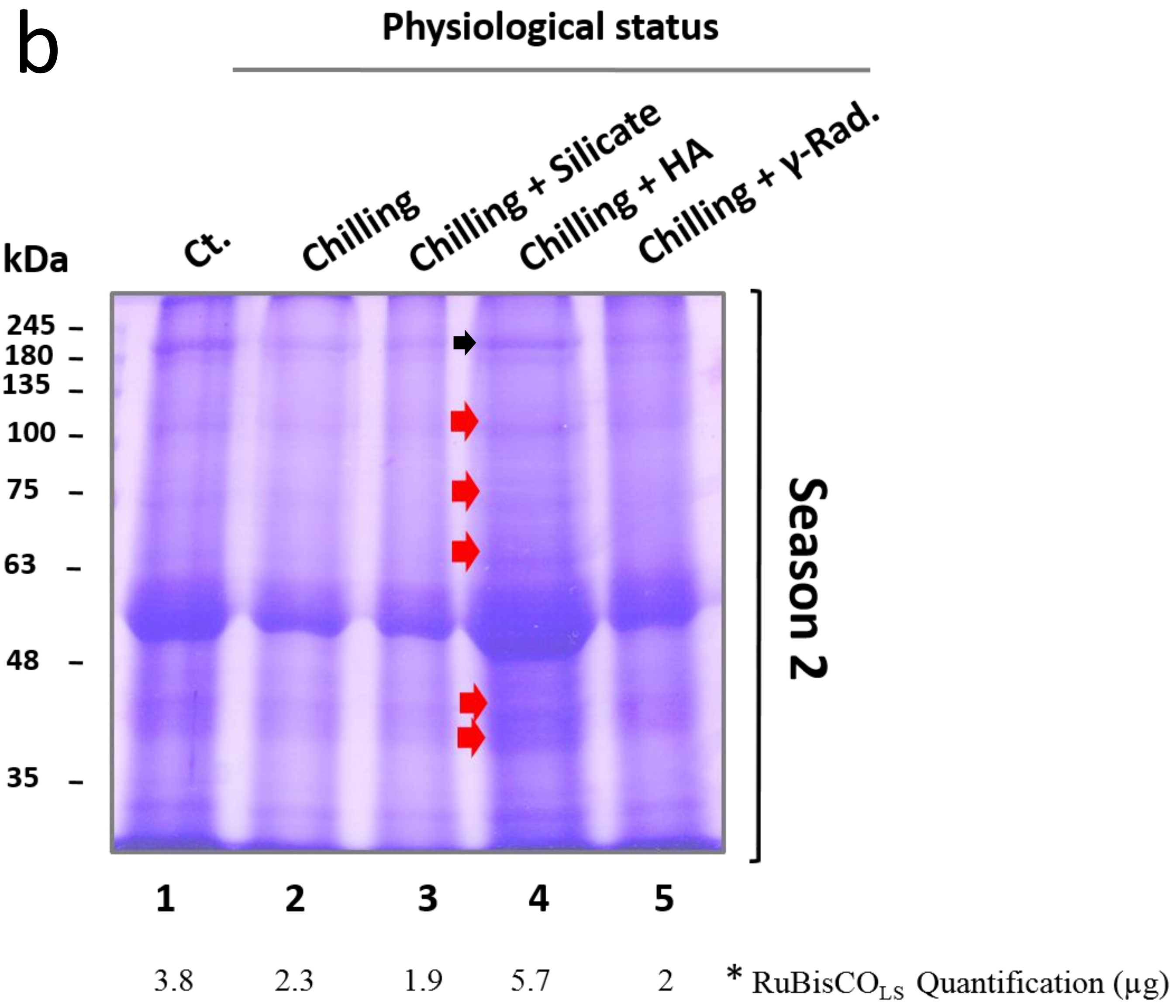
b
Physiological status
Ct.
Chilling
Chilling + Silicate
Chilling + HA
Chilling + γ-Rad.
kDa
245
180
135
100
75
63
48
35
Season 2
1
2
3
4
5
3.8
2.3
1.9
5.7
2
* RuBisCO$_{LS}$ Quantification (µg)

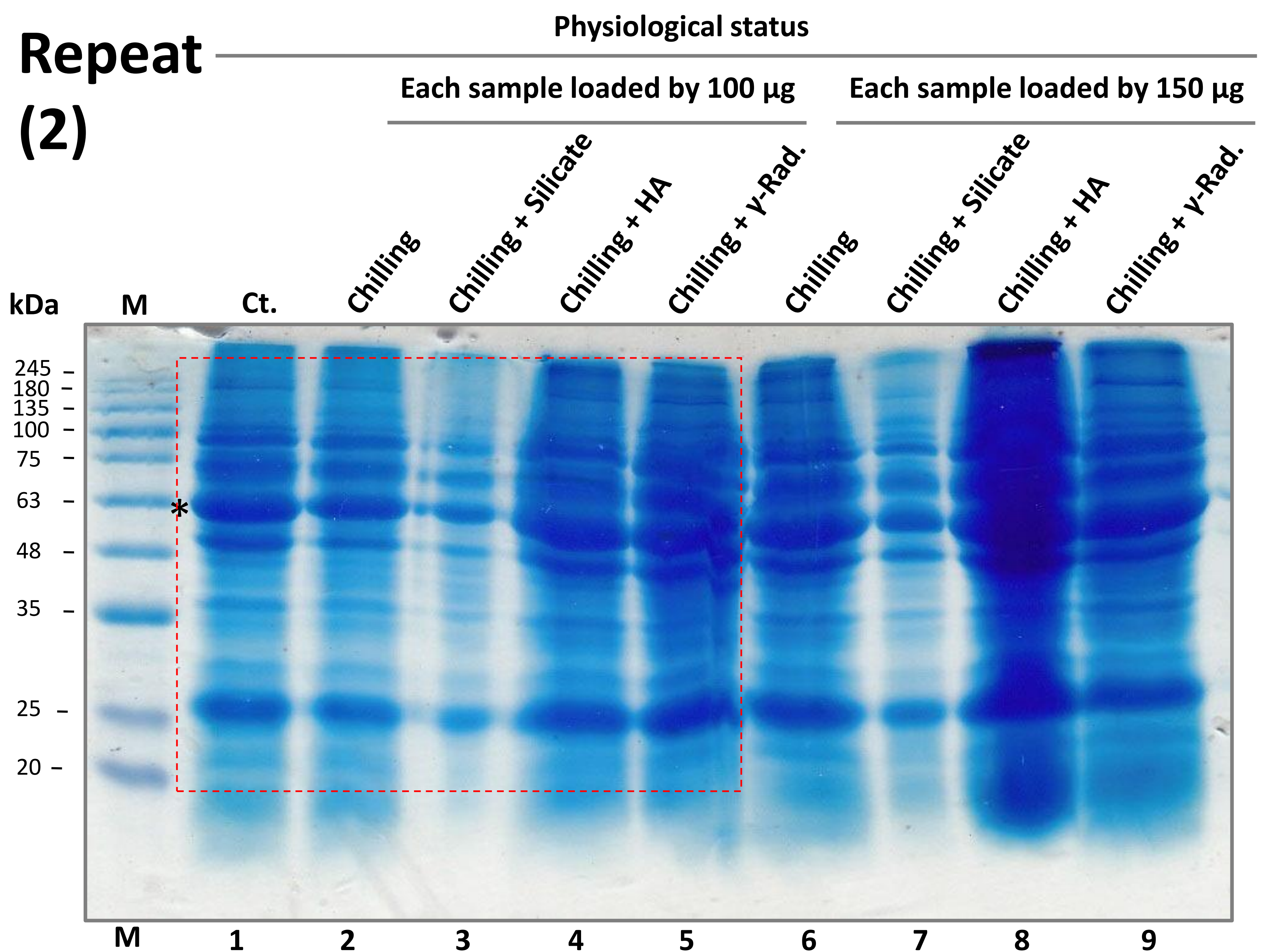

Repeat (2)
Physiological status
Each sample loaded by 100 µg
Each sample loaded by 150 µg
kDa
M
Ct.
Chilling
Chilling + Silicate
Chilling + HA
Chilling + γ-Rad.
Chilling
Chilling + Silicate
Chilling + HA
Chilling + γ-Rad.
245
180
135
100
75
63
48
35
25
20
*
M
1
2
3
4
5
6
7
8
9

Supplementary Figure 2

Construction steps of the (0, 1) Binary matrix

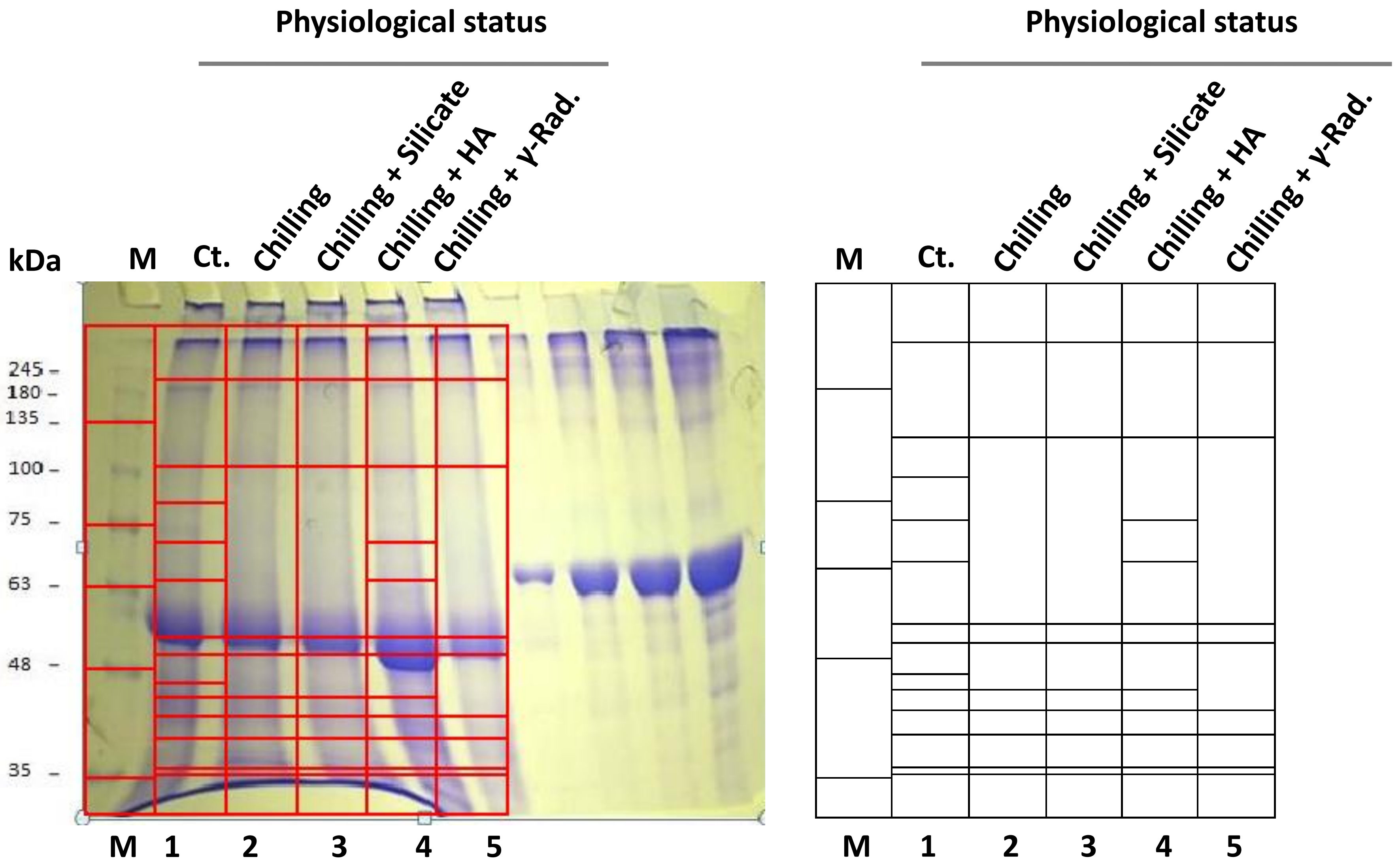

Physiological status
kDa
M
Ct.
Chilling
Chilling + Silicate
Chilling + HA
Chilling + γ-Rad.
245
180
135
100
75
63
48
35
M
1
2
3
4
5
Physiological status
M
Ct.
Chilling
Chilling + Silicate
Chilling + HA
Chilling + γ-Rad.
M
1
2
3
4
5

Rate of flow (Rf) of separated polypeptides

RF					
Marker	1	2	3	4	5
0.20	0.11	0.11	0.11	0.11	0.11
0.41	0.29	0.29	0.29	0.29	0.29
0.53	0.36	0.64	0.64	0.44	0.64
0.70	0.44	0.67	0.67	0.52	0.67
0.93	0.52	0.76	0.76	0.64	0.80
-----	0.64	0.80	0.80	0.67	0.85
-----	0.67	0.85	0.85	0.76	0.91
-----	0.73	0.91	0.91	0.80	0.92
-----	0.76	0.92	0.92	0.85	-----
-----	0.80	-----	-----	0.91	-----
-----	0.85	-----	-----	0.92	-----
-----	0.91	-----	-----	-----	-----
-----	0.92	-----	-----	-----	-----

Molecular weights of separated polypeptides

MD					
M	1	2	3	4	5
121	142	142	142	142	142
83	103	103	103	103	103
66	90	55	55	78	55
49	78	52	52	68	52
33	68	44	44	55	41
-----	55	41	41	52	38
-----	52	38	38	44	34
-----	46	34	34	41	33
-----	44	33	33	38	-----
-----	41	-----	-----	34	-----
-----	38	-----	-----	33	-----
-----	34	-----	-----	-----	-----
-----	33	-----	-----	-----	-----

Band scoring of separated polypeptides

0 / 1							
M	1	2	3	4	5	Frequency	Polymorphism
142	1	1	1	1	1	1	Monomorphic
103	1	1	1	1	1	1	Monomorphic
90	1	0	0	0	0	0	Unique
78	1	0	0	1	0	0	Polymorphic
68	1	0	0	1	0	0	Polymorphic
55	1	1	1	1	1	1	Monomorphic
52	1	1	1	1	1	1	Monomorphic
46	1	0	0	0	0	0	Unique
44	1	1	1	1	0	1	Polymorphic
41	1	1	1	1	1	1	Monomorphic
38	1	1	1	1	1	1	Monomorphic
34	1	1	1	1	1	1	Monomorphic
33	1	1	1	1	1	1	Monomorphic

Supplementary Figure 3

Western blot original individual panels

α-Actin

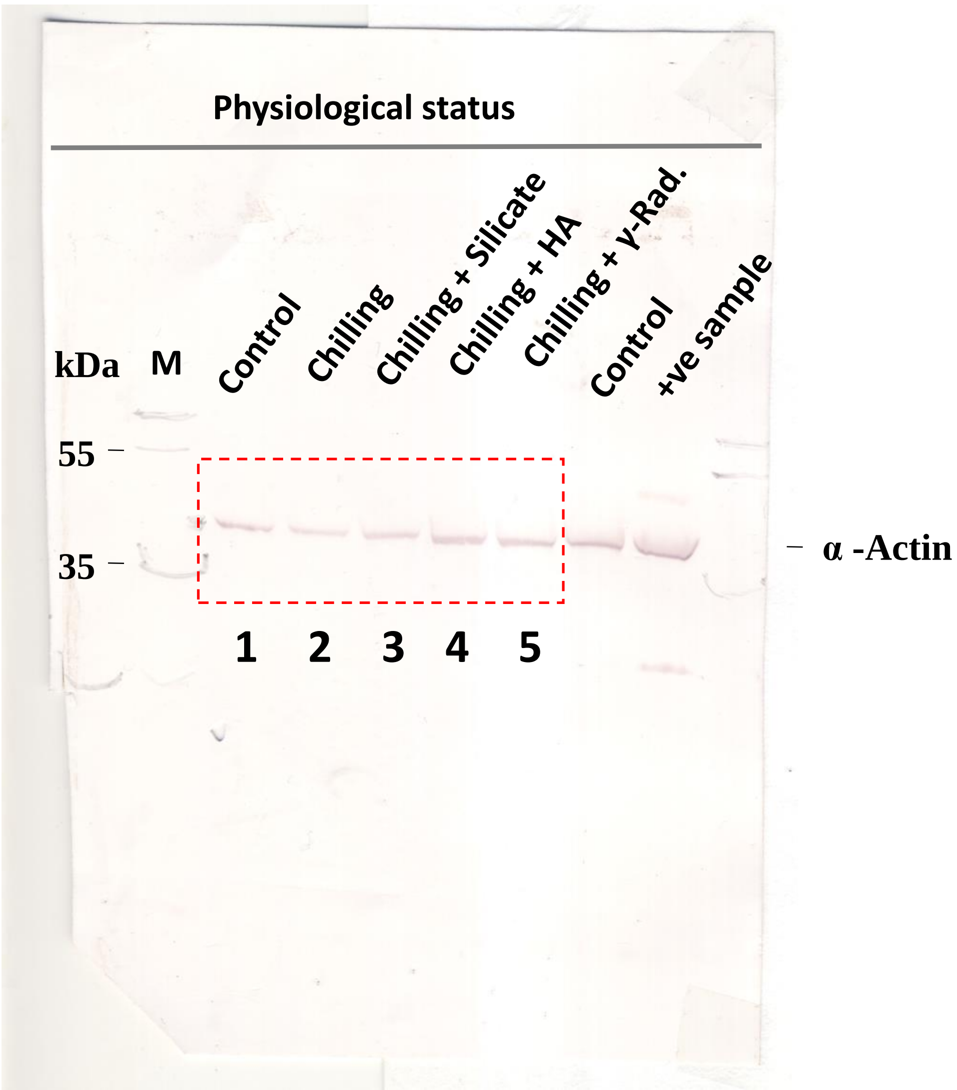

Physiological status
kDa
M
Control
Chilling
Chilling + Silicate
Chilling + HA
Chilling + γ-Rad.
Control
+ve sample
55
35
α -Actin
1
2
3
4
5

α-Toc75

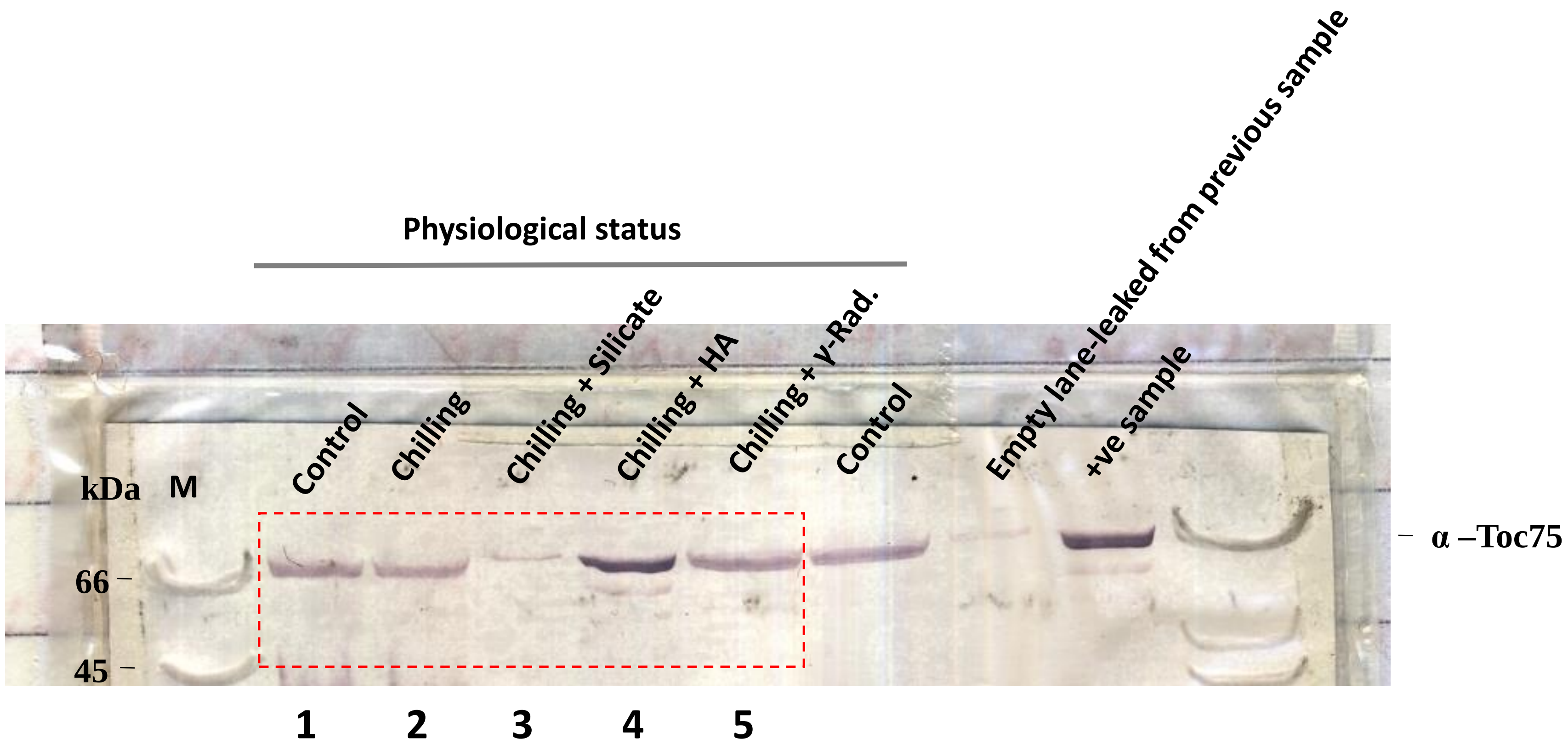
Physiological status
Control
Chilling
Chilling + Silicate
Chilling + HA
Chilling + γ-Rad.
Control
Empty lane-leaked from previous sample
+ve sample
kDa
M
66
45
α –Toc75
1
2
3
4
5

α-Toc34

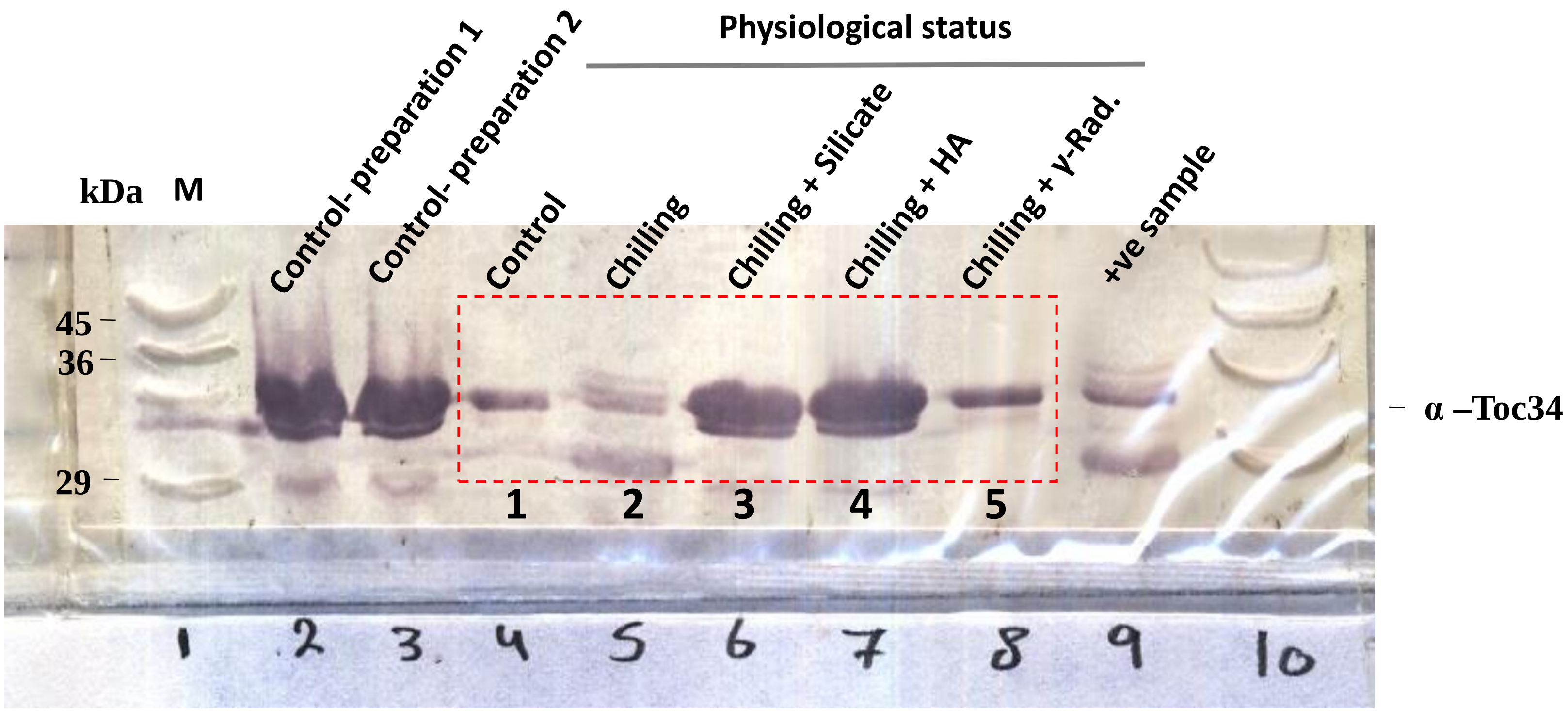

Physiological status
kDa
M
Control- preparation 1
Control- preparation 2
Control
Chilling
Chilling + Silicate
Chilling + HA
Chilling + γ-Rad.
+ve sample
45
36
29
α –Toc34
1
2
3
4
5
1 2 3 4 5 6 7 8 9 10

α-eHSP70

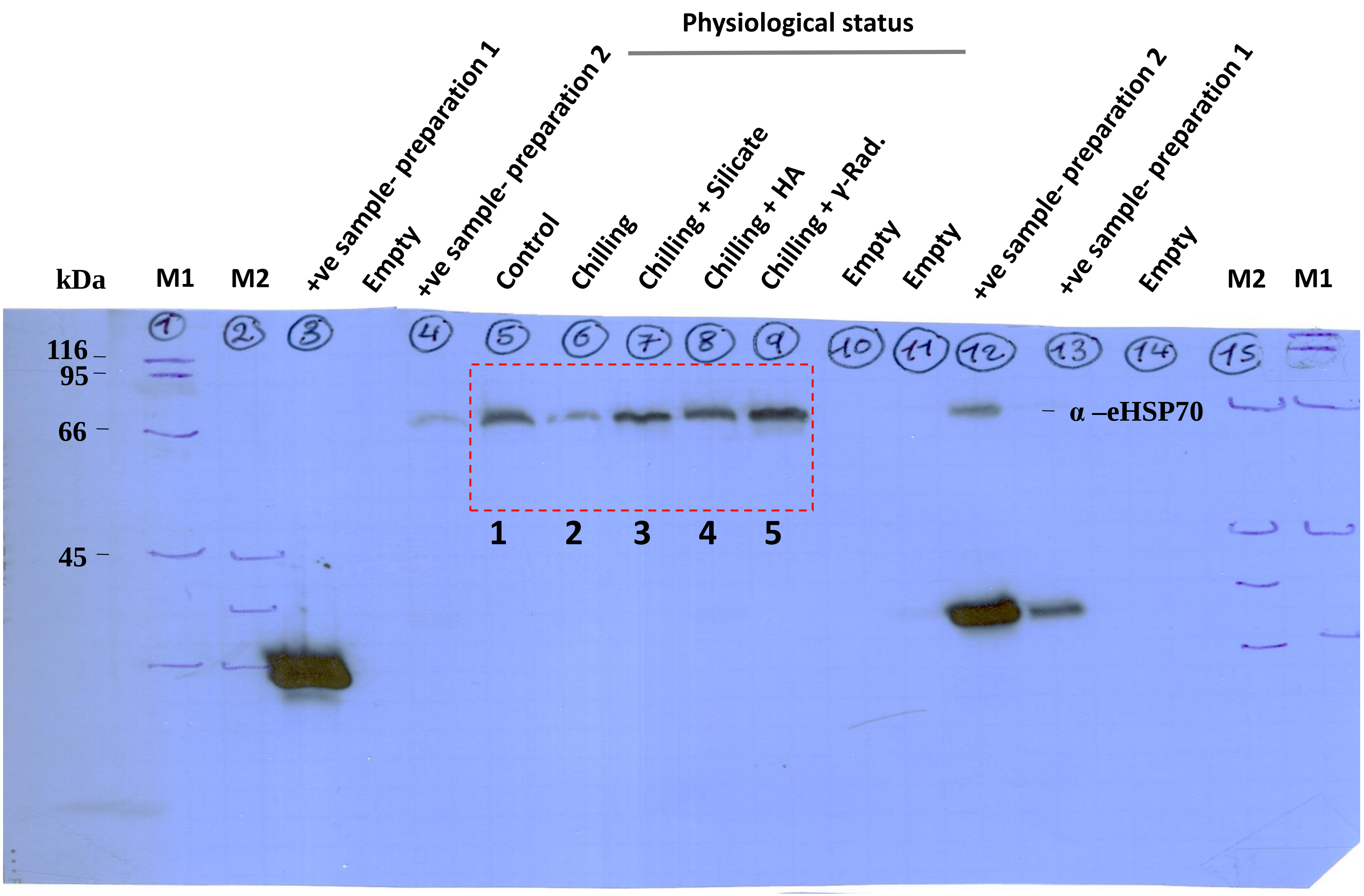
Physiological status
kDa
M1
M2
+ve sample- preparation 1
Empty
+ve sample- preparation 2
Control
Chilling
Chilling + Silicate
Chilling + HA
Chilling + γ-Rad.
Empty
Empty
+ve sample- preparation 2
+ve sample- preparation 1
Empty
M2
M1
116
95
66
45
1
2
3
4
5
α –eHSP70

Supplementary Figure 4

HDN-PAGE

Original and unprocessed version

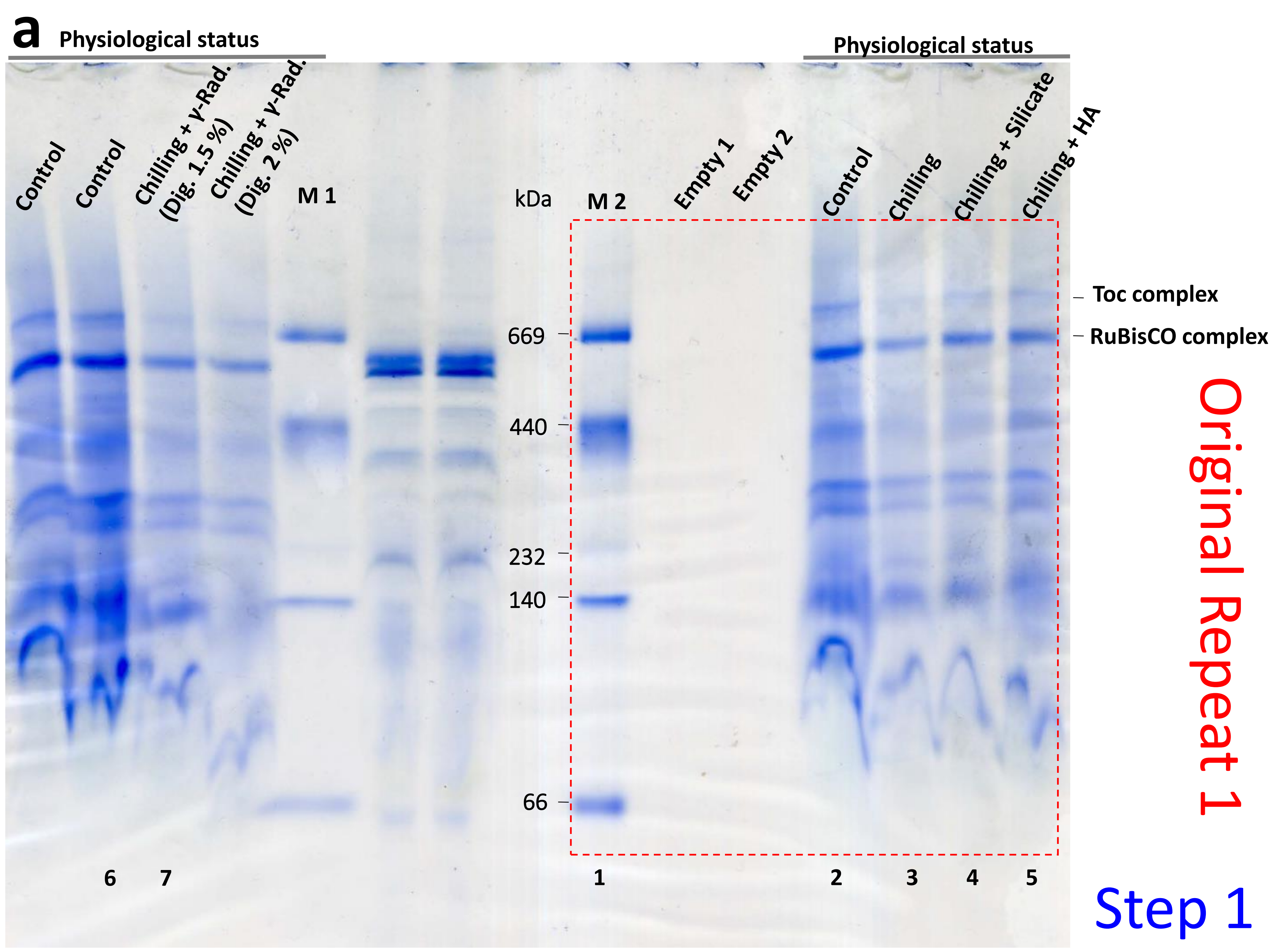

a
Physiological status
Control
Control
Chilling + γ-Rad. (Dig. 1.5 %)
Chilling + γ-Rad. (Dig. 2 %)
M 1
kDa
M 2
Empty 1
Empty 2
Physiological status
Control
Chilling
Chilling + Silicate
Chilling + HA
Toc complex
RuBisCO complex
669
440
232
140
66
Original Repeat 1
6
7
1
2
3
4
5
Step 1

1- Removing Empty 1 and Empty 2 lanes

2- M2 is placed to be adjacent beside control sample (at right hand side of the gel)

Original Repeat 1

Step 2

1- Removing lanes 6 and 7 from the left hand side of the gel.

2- Cropped lanes were flipped horizontally.

3- Lanes 6 and 7 were placed beside other lanes from 1-5.

Original Repeat 1

Step 3

Processed version

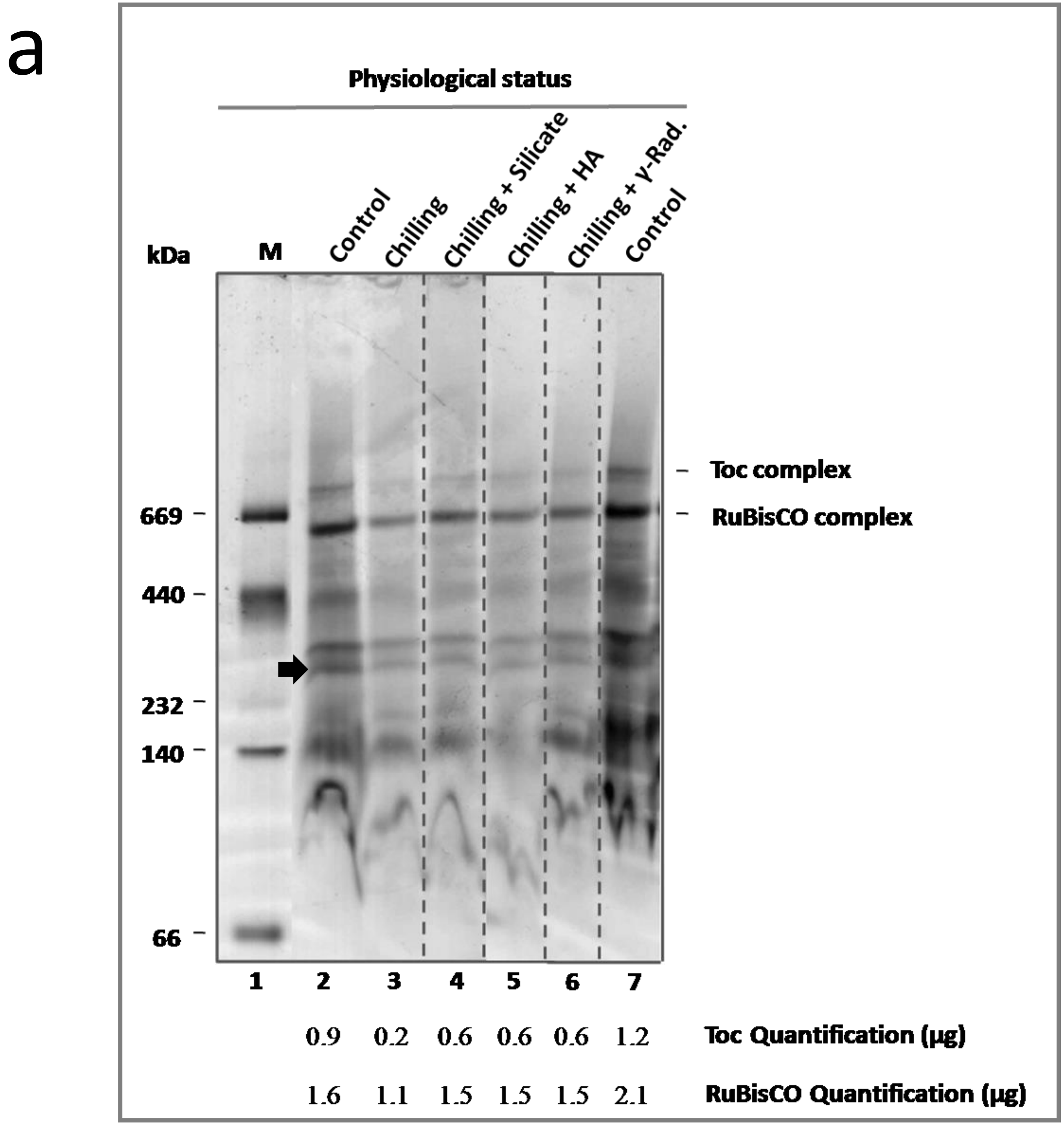
a
Physiological status
kDa
M
Control
Chilling
Chilling + Silicate
Chilling + HA
Chilling + γ-Rad.
Control
669
440
232
140
66
Toc complex
RuBisCO complex
1 2 3 4 5 6 7
0.9 0.2 0.6 0.6 0.6 1.2
Toc Quantification (µg)
1.6 1.1 1.5 1.5 1.5 2.1
RuBisCO Quantification (µg)

Original and unprocessed version

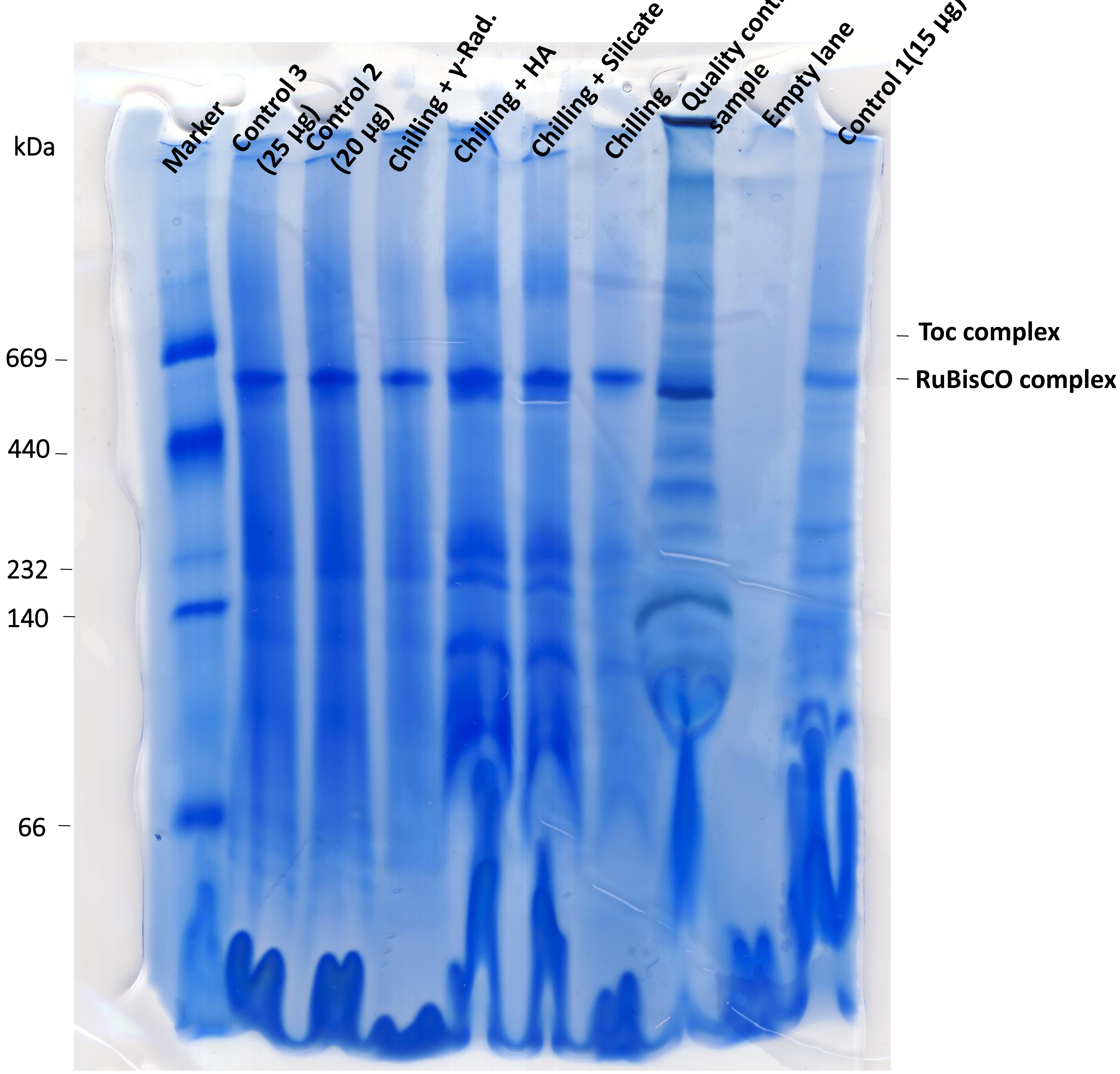
kDa
669
440
232
140
66
Marker
Control 3 (25 µg)
Control 2 (20 µg)
Chilling + γ-Rad.
Chilling + HA
Chilling + Silicate
Chilling
Quality cont
sample
Empty lane
Control 1(15 µg
Toc complex
RuBisCO complex

Repeat 2

Processed version

b

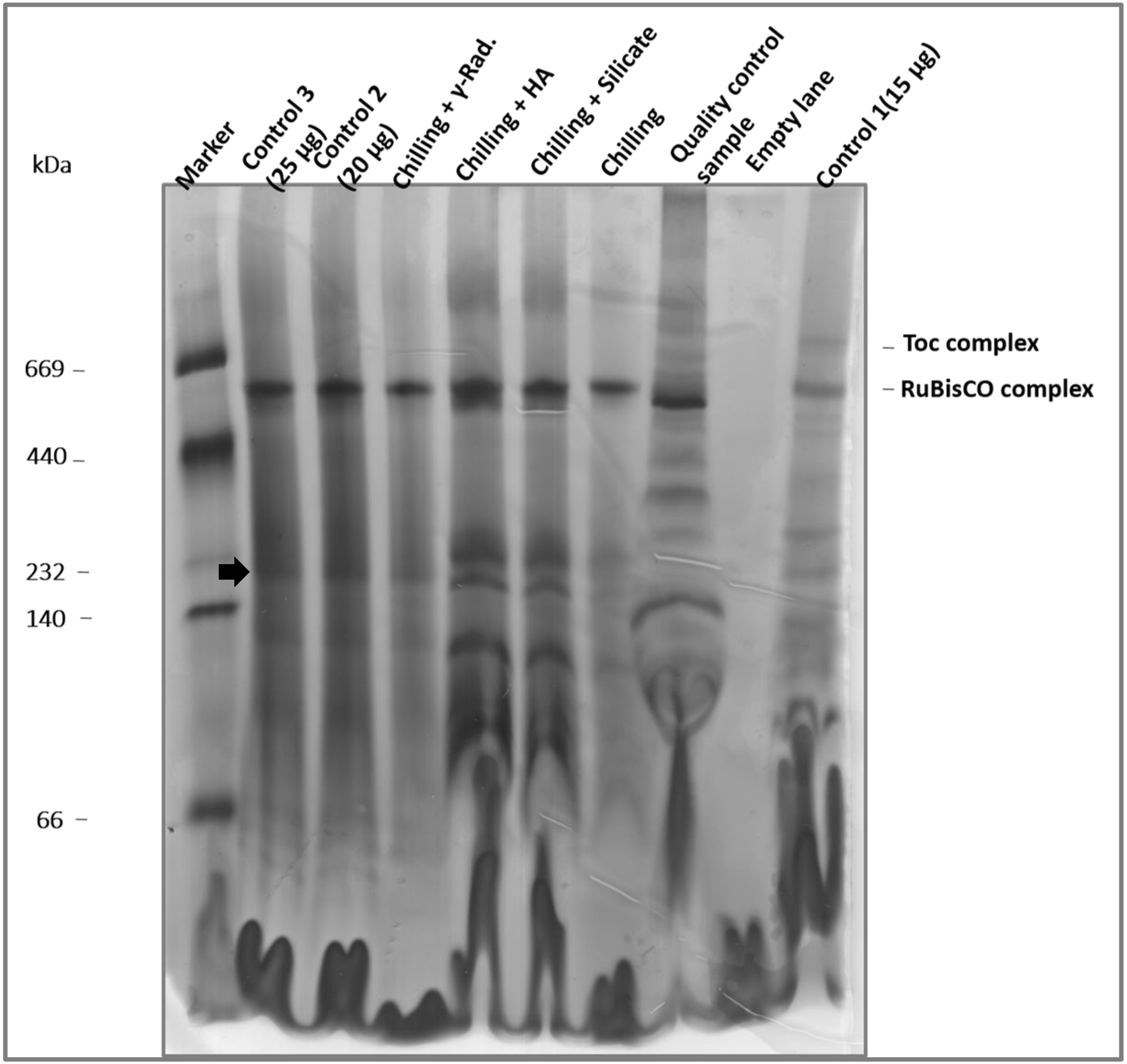
kDa
Marker
Control 3 (25 µg)
Control 2 (20 µg)
Chilling + γ-Rad.
Chilling + HA
Chilling + Silicate
Chilling
Quality control sample
Empty lane
Control 1(15 µg)
669
440
232
140
66
Toc complex
RuBisCO complex
