# Supplementary material for: Metabolic activities and molecular investigations of the ameliorative impact of some growth biostimulators on chilling-stressed coriander (Coriandrum sativum L.) plant
Source: BMC Plant Biol. 2021 Aug 7;21:361. doi: 10.1186/s12870-021-03021-6 (PMC8349021; doi:10.1186/s12870-021-03021-6)
Supplement: Supplementary file 3 — Additional file 3: Supplementary Table 1. Correlation matrix linking the interaction between the application of biostimulants applied on the chilling-stressed on coriander (Coriandrum sativum L.) seeds pre- soaked in 80 mM pot. Silicate, 50 mg l− 1 humic acid or soaked in water after exposure to γ-rays (50 Gy) and the improvement in both the photosynthetic pigments (μg/g D. wt. in coriander leaves) and carbohydrate contents (g/100 g D. wt.) at the vegetative and flowering stages. *. Correlation is significant at the 0.05 level (2-tailed). **. Correlation is significant at the 0.01 level (2-tailed). [file 12870_2021_3021_MOESM3_ESM.docx]

**Supplementary Table 1.** Correlation matrix linking the interaction between the application of biostimulants applied on the chilling-stressed on coriander (*Coriandrum sativum* L*.*) seeds pre- soaked in 80 mM pot. silicate, 50 mg l^-1^ humic acid or soaked in water after exposure to γ-rays (50 Gy) and the improvement in both the photosynthetic pigments (µg/g D. wt. in coriander leaves) and carbohydrate contents (g/100g D. wt.) at the vegetative and flowering stages. *. Correlation is significant at the 0.05 level (2-tailed). **. Correlation is significant at the 0.01 level (2-tailed).

|  | Shoot length | No. of leaves | No. of branches | leaves area | F. wt. of shoot | D. wt. of shoot | Root length | F. wt. of root | D. wt. of root | No. of inflorescences | Chl a | Chl b | Soluble sugars | Polysaccharides | Total carbohydrates |
| --- | --- | --- | --- | --- | --- | --- | --- | --- | --- | --- | --- | --- | --- | --- | --- |
| shoot length | 1 | .746^*^ | .925^**^ | .873^**^ | .872^**^ | .885^**^ | .894^**^ | .884^**^ | .911^**^ | .800^*^ | .929^**^ | .919^**^ | .345 | .903^**^ | .872^**^ |
| No. of leaves | .746^*^ | 1 | .889^**^ | .944^**^ | .751^*^ | .759^*^ | .718^*^ | .648 | .704 | .890^**^ | .900^**^ | .743^*^ | .447 | .894^**^ | .879^**^ |
| No. of branches | .925^**^ | .889^**^ | 1 | .927^**^ | .881^**^ | .923^**^ | .816^*^ | .888^**^ | .910^**^ | .876^**^ | .950^**^ | .847^**^ | .296 | .893^**^ | .855^**^ |
| Leave area | .873^**^ | .944^**^ | .927^**^ | 1 | .864^**^ | .857^**^ | .869^**^ | .807^*^ | .861^**^ | .929^**^ | .930^**^ | .834^*^ | .499 | .921^**^ | .913^**^ |
| F. wt. of shoot | .872^**^ | .751^*^ | .881^**^ | .864^**^ | 1 | .987^**^ | .936^**^ | .936^**^ | .866^**^ | .942^**^ | .845^**^ | .841^**^ | .525 | .846^**^ | .848^**^ |
| D. wt. of shoot | .885^**^ | .759^*^ | .923^**^ | .857^**^ | .987^**^ | 1 | .901^**^ | .964^**^ | .895^**^ | .916^**^ | .858^**^ | .823^*^ | .436 | .829^*^ | .819^*^ |
| Root length | .894^**^ | .718^*^ | .816^*^ | .869^**^ | .936^**^ | .901^**^ | 1 | .867^**^ | .810^*^ | .872^**^ | .829^*^ | .812^*^ | .673 | .863^**^ | .887^**^ |
| F. wt. of root | .884^**^ | .648 | .888^**^ | .807^*^ | .936^**^ | .964^**^ | .867^**^ | 1 | .951^**^ | .812^*^ | .799^*^ | .774^*^ | .339 | .735^*^ | .719^*^ |
| D. wt. of root | .911^**^ | .704 | .910^**^ | .861^**^ | .866^**^ | .895^**^ | .810^*^ | .951^**^ | 1 | .795^*^ | .831^*^ | .818^*^ | .196 | .754^*^ | .714^*^ |
| No of inflorescences | .800^*^ | .890^**^ | .876^**^ | .929^**^ | .942^**^ | .916^**^ | .872^**^ | .812^*^ | .795^*^ | 1 | .862^**^ | .825^*^ | .540 | .885^**^ | .886^**^ |
| Chl a | .929^**^ | .900^**^ | .950^**^ | .930^**^ | .845^**^ | .858^**^ | .829^*^ | .799^*^ | .831^*^ | .862^**^ | 1 | .927^**^ | .435 | .978^**^ | .954^**^ |
| Chl b | .919^**^ | .743^*^ | .847^**^ | .834^*^ | .841^**^ | .823^*^ | .812^*^ | .774^*^ | .818^*^ | .825^*^ | .927^**^ | 1 | .358 | .931^**^ | .900^**^ |
| Soluble sugars | .345 | .447 | .296 | .499 | .525 | .436 | .673 | .339 | .196 | .540 | .435 | .358 | 1 | .549 | .653 |
| Polysaccharides | .903^**^ | .894^**^ | .893^**^ | .921^**^ | .846^**^ | .829^*^ | .863^**^ | .735^*^ | .754^*^ | .885^**^ | .978^**^ | .931^**^ | .549 | 1 | .992^**^ |
| Total carbohydrates | .872^**^ | .879^**^ | .855^**^ | .913^**^ | .848^**^ | .819^*^ | .887^**^ | .719^*^ | .714^*^ | .886^**^ | .954^**^ | .900^**^ | .653 | .992^**^ | 1 |
